# Mapping the Synthetic Dosage Lethality Network of *CDK1/CDC28*

**DOI:** 10.1534/g3.117.042317

**Published:** 2017-04-18

**Authors:** Christine Zimmermann, Ignacio Garcia, Manja Omerzu, Pierre Chymkowitch, Beibei Zhang, Jorrit M. Enserink

**Affiliations:** * Department of Molecular Cell Biology, Institute for Cancer Research, The Norwegian Radium Hospital, Montebello, 0379 Oslo, Norway; † Section for Biochemistry and Molecular Biology, Faculty of Mathematics and Natural Sciences, University of Oslo, 0371, Norway

**Keywords:** Cdc28, Cdk1, cell cycle, synthetic lethality, Mutant Screen Report

## Abstract

Cdk1 (Cdc28 in yeast) is a cyclin-dependent kinase (CDK) essential for cell cycle progression and cell division in normal cells. However, CDK activity also underpins proliferation of tumor cells, making it a relevant study subject. While numerous targets and processes regulated by Cdc28 have been identified, the exact functions of Cdc28 are only partially understood. To further explore the functions of Cdc28, we systematically overexpressed ∼4800 genes in wild-type (WT) cells and in cells with artificially reduced Cdc28 activity. This screen identified 366 genes that, when overexpressed, specifically compromised cell viability under conditions of reduced Cdc28 activity. Consistent with the crucial functions of Cdc28 in cell cycle regulation and chromosome metabolism, most of these genes have functions in the cell cycle, DNA replication, and transcription. However, a substantial number of genes control processes not directly associated with the cell cycle, indicating that Cdc28 may also regulate these processes. Finally, because the dataset was enriched for direct Cdc28 targets, the results from this screen will aid in identifying novel targets and process regulated by Cdc28.

CDKs drive the cell cycle in eukaryotic cells. A single CDK, Cdc28, is necessary and sufficient for cell cycle regulation in the budding yeast *Saccharomyces cerevisiae* (Mendenhall and Hodge 1998; Enserink and Kolodner 2010), although many of its functions are supported by the nonessential CDK Pho85, and there exists substantial cross talk between these kinases in the regulation of cell cycle-related processes (Huang *et al.* 2007). Cdc28 is activated by its cyclin partners, which are differentially expressed throughout the cell cycle. Cyclin–Cdc28 complexes coordinate the cell cycle by phosphorylating specific proteins involved in DNA replication and repair, telomere homeostasis, cell growth and morphogenesis, lipid synthesis, formation of the mitotic spindle, and transcriptional programs (Enserink and Kolodner 2010). Cdc28 is a proline-directed kinase that preferentially phosphorylates the consensus sequence S/T-P-x-K/R (where x is any amino acid), although it also phosphorylates the minimal consensus sequence S/T-P (Moreno and Nurse 1990).

Aberrant CDK activity underlies uncontrolled proliferation of tumor cells (Hunter and Pines 1994), which is why it is important to study its functions. However, while Cdc28 is one of the best studied kinases with a well-described repertoire of substrates (Enserink and Kolodner 2010), its exact molecular functions are not fully understood. An important technological improvement was the development of the engineered *cdc28-as1* allele (Bishop *et al.* 2000), which encodes a form of Cdc28 in which the gatekeeper phenylalanine at position 88 is replaced with a much smaller glycine residue. This creates an enlarged ATP binding pocket that can accommodate the bulky, nonhydrolyzable ATP analog 1-NM-PP1 (Bishop *et al.* 2000). Treatment of *cdc28-as1* mutants with the cell-permeable 1-NM-PP1 results in specific inhibition of Cdc28 (Bishop *et al.* 2000). The effect of 1-NM-PP1 on cell cycle progression of *cdc28-as1* mutants is dose-dependent; at lower doses (≤500 nM) it causes a delay or arrest with 2N DNA content and large hyperpolarized buds, whereas higher doses (5000 nM) arrest cell cultures as a mix of unbudded G1 cells and large-budded M phase cells. However, it should be noted that the gene product of *cdc28-as1*, Cdc28-as1, has a 10-fold reduction in ATP-binding affinity and a sixfold reduction in maximum ATP turnover rate (Bishop *et al.* 2000). As a consequence, the *cdc28-as1* mutant is hypomorphic and has a 20% longer doubling time in liquid culture (Bishop *et al.* 2000), although this increased doubling time is not obvious in spot assays (Zimmermann *et al.* 2011).

Despite its hypomorphic nature, the *cdc28-as1* allele is a powerful tool that has been used in multiple studies to identify novel functions and targets of Cdc28; for instance, it has been used to identify Cdc28-dependent phosphorylations *in vivo* using a SILAC-based phosphoproteomics approach (Holt *et al.* 2009). Another interesting feature of Cdc28-as1 is that it can use N^6^-(benzyl) ATP as an ATP source, which cannot be used by other kinases due its bulkiness. One drawback of N^6^-(benzyl) ATP is that it is not cell permeable, restricting its use to *in vitro* experiments. Nevertheless, a screen for proteins that are directly phosphorylated by Cdc28 in whole-cell extracts identified ∼200 Cdc28 substrates, several of which were confirmed to be phosphorylated *in vivo* in a Cdc28-dependent manner (Ubersax *et al.* 2003).

Significant insight into the function and regulation of Cdc28 has also come from classic dosage suppression screens, in which genes were identified that complement the slow growth phenotype of temperature-sensitive *cdc28* alleles. For example, these screens identified several cyclins as well as Cks1, which is a subunit of the Cdc28 holoenzyme (Hadwiger *et al.* 1989a,b; Surana *et al.* 1991). Such classic genetic screens can be relatively laborious, often involving the organization of suppressors in complementation groups followed by mapping and identification of the genes responsible for suppression of the mutant phenotype. More recently, automated high-throughput genetic screens have been developed to systematically interrogate the genetic landscape of cells. One commonly used method is synthetic genetic array (SGA; Tong *et al.* 2001). Most SGA screens use synthetic lethality as a readout. The concept of synthetic lethality was first developed in *Drosophila* to describe the observation that the combination of two mutations results in a significantly worse fitness defect than either single mutation alone (Dobzhansky 1946). Genes with synthetic lethal interactions often function in parallel nonessential pathways that control an essential cellular process (Dixon *et al.* 2009). Therefore, synthetic lethality can be used to identify new regulators of specific cellular processes, or assign new functions to genes (Enserink 2012). For instance, we recently reported an SGA screen in which we used low doses of 1-NM-PP1 that partially reduced the growth of *cdc28-as1* single mutants, and screened for deletion mutants that aggravated the growth defect of the *cdc28-as1* mutant. This screen identified 107 genes that are essential for maintaining cell viability under conditions of reduced Cdc28 activity, and revealed several cellular processes that were not previously known to affect the cell cycle (Zimmermann *et al.* 2011). Follow-up studies revealed novel functions of Cdc28; for instance, we have shown that Cdc28 has a function in stimulating the transcription of housekeeping genes, which is important for cell homeostasis (Chymkowitch *et al.* 2012).

Another high-throughput method to identify novel regulators of cellular processes is synthetic dosage lethality (SDL) screening, which is based on the idea that increasing the levels of a protein results in a significantly worse fitness defect in a mutant strain than in an otherwise WT strain (Kroll *et al.* 1996). Because it induces specific regulatory imbalances in pathways that regulate critical cellular pathways, systematic gene overexpression has revealed novel regulatory pathways and phenotypes (Kroll *et al.* 1996; Measday *et al.* 2005; Sopko *et al.* 2006). For example, recently, SDL screening was applied to interrogate the yeast kinome using 92 kinases as a query, revealing potential novel functions for many kinases (Sharifpoor *et al.* 2012). SDL datasets tend to be enriched for kinase substrates, enabling the identification of key downstream components of cell signaling pathways (Sopko *et al.* 2006; Sharifpoor *et al.* 2012). Thus, SDL screening is a useful tool for the identification of novel kinase functions and kinase substrates, and in the present study we performed an SDL screen to probe the functions of Cdc28.

## Materials and Methods

### Yeast strains

The WT strain (BY4741) and the isogenic *cdc28-as1* query strain (JEY4250) have been published previously (Brachmann *et al.* 1998; Zimmermann *et al.* 2011).

### Genetic screen

Strains BY4741 and JEY4250 were systematically transformed with the arrayed yeast movable ORF library (Gelperin *et al.* 2005), and grown overnight in liquid dropout media lacking uracil (to select for the plasmid library) and supplemented with 2% glucose. Overnight cultures were then pinned onto synthetic dropout plates lacking uracil and containing either 2% glucose or 2% galactose, and supplemented with either DMSO, 50 nM 1-NM-PP1, or 100 nM 1-NM-PP1. Plates were then incubated at 30° for 2–3 d and photographed. Phenotypes that were scored were either aggravation of the growth defect of the *cdc28-as1* mutant with respect to the WT control, or relatively improved growth compared with a *cdc28-as1* mutant overexpressing *YEL074W*, which is a dubious ORF with no known genetic interactions (overexpression of *YEL074W* does not affect growth of either WT cells or *cdc28-as1* mutants). The entire screen was repeated once using essentially the same set-up, although we spotted four different dilutions (1:10, 1:30, 1:100, and 1:300) of overnight cultures onto the selection plates instead of pinning WT cells and *cdc28-as1* mutants at one single concentration.

### STRING analysis

STRING analysis (Szklarczyk *et al.* 2015) was carried out at highest confidence (0.900) using the query proteins only and using default settings, with the following exceptions: Active interaction sources: “Experiments,” “Databases.” Network edges: “Evidence.”

### Gene Ontology (GO) analysis and visualization

GO analysis and visualization of the GO network was performed using Metascape (http://metascape.org; Tripathi *et al.* 2015) using parameters specific for *S. cerevisiae*. Additional analysis of the overrepresented biological processes of the SDL network was performed using GO Slim mapper at the SGD database using the term “Yeast GO-Slim: Process,” selecting all terms present in the database.

### Comparison with other datasets

High- and low-throughput genetic interaction data (synthetic lethality, negative and positive genetic interactions, dosage rescue, and dosage lethality) were obtained from BioGRID (Chatr-Aryamontri *et al.* 2017). Physical interactions with the Cdc28 holoenzyme (Cln1-3, Clb1-6, Cdc28, and Cks1; both high- and low-throughput data) were also derived from BioGRID. Because physical interaction data from high-throughput studies can consist of up to 50% false positives (von Mering *et al.* 2002; Patil and Nakamura 2005), we only included physical interactions that were observed at least twice per Cdc28 holoenzyme subunit. Data regarding *in vitro* phosphorylation by Cdc28 was obtained from Ubersax *et al.* (2003) (185 proteins; cut-off for inclusion: *P* score ≥ 2) and Ptacek *et al.* (2005) (67 proteins). *In vivo* Cdc28-dependent phosphorylation data were compiled from high- and low-throughput data derived from PhosphoGRID (Sadowski *et al.* 2013) and Phosphopep (Bodenmiller and Aebersold 2011; Bodenmiller *et al.* 2010), from Holt *et al.* (2009) (cut-off for inclusion: log2 ≤ 1), and from publications that appeared to be absent from PhosphoGRID and Phosphopep (Han *et al.* 2005; Liu *et al.* 2011; Tao *et al.* 2011; Yaglom *et al.* 1995; Zhu *et al.* 2010). The list of known Cdc28 substrates was based on our previous compilation of Cdc28 targets (Enserink and Kolodner 2010), which was updated with information from recent literature.

### RNA extraction, reverse transcription, and qPCR

Log phase *cdc28-as1* cells were treated with 500 nM of 1-NM-PP1 in YPD. After 4 hr of treatment, cell cycle arrest in M phase was confirmed by flow cytometry (see below). *CLN3* expression was induced by switching cells to galactose-containing medium for 4 hr. Then, RNA was isolated using the RNeasy Mini Kit (QIAGEN) and equal amounts of RNA were reverse transcribed using the QuantiTect Reverse Transcription Kit (QIAGEN). qPCRs were performed using the Power SYBR Green PCR Master Mix (Applied Biosystems) and the StepOnePlus real-time PCR system (Applied Biosystems).

### Primer sequences

Primer sequences used are as follows: *CLN2*: 5′-TATCCCAGGATAGTGATGCCACTG-3′ and 5′-TCTAAGTAAGTCGTACTGCCACGC-3′; *CLN3*: 5′-CAAAGAGCGCTACGGTTTCATCTG-3′ and 5′-TGGAGAGGATGAAGATGAGGTTGG-3′; *CLB6*: 5′-CATCACTTGCCTGTTCATTGCCTG-3′ and 5′-AGCTCAGCCTTCCTAATTCCTTCG-3′; and *ACT1*: 5′-TGAGGAGCACCCTTGCTTGT-3′ and 5′-TCTTCTCACGGTTGGATTTGG-3′.

### Cell cycle analysis by flow cytometry

*cdc28-as1* mutant cells were treated as described under *RNA extraction*, *reverse transcription*, *and qPCR* before being processed for cell cycle analysis by flow cytometry as previously described (Enserink *et al.* 2009). Briefly, cells were fixed in 70% EtOH (vol/vol), centrifuged, and resuspended in 50 mM sodium citrate + 0.2 µg/µl of RNAse A. After 2 hr at 37°, cells were centrifuged and resuspended in 50 mM sodium citrate + 5 mg/ml of pepsin. After 30 min at 37°, cells were centrifuged and resuspended in 50 mM sodium citrate + 2 µg/ml of propidium iodine and analyzed by flow cytometry using a BD FACSCalibur instrument. Cell cycle profiles were analyzed with FlowJo software.

### Quantification of spindle alignment and morphology

To determine the effect of the overexpression of different genes on mitotic spindle alignment and morphology, WT (JEY5235) and *cdc28-as1* (JEY5239) strains expressing the Tub1-GFP fusion protein were transformed with the plasmids harboring *YEL074W* (control), *CIN8*, *FIN1*, *KAR9*, and *SPC97*. Cultures were grown to log phase in synthetic dropout media without uracil containing 2% glucose, followed by incubation in dropout medium containing 2% galactose for 4 hr, after which cells were imaged in a Zeiss Axioplan2 microscope coupled to a Zeiss AxioCam HRC camera. To estimate the mitotic spindle orientation, the angle between the bud axis and the spindle axis was measured for each large-budded cell and the spindle was counted as misaligned when this angle was bigger than 45°. The percentage of aberrant mitotic spindles was measured by comparison with the spindle morphology of WT cells. All image processing and quantification was performed in ImageJ.

### Quantification of nuclear localization of Whi5-GFP

Whi5-GFP-expressing cells were grown overnight in dropout medium lacking uracil, centrifuged, and resuspended in YPD. Cells were then arrested in M phase using 15 µg/ml nocodazole for 2.5 hr and M phase arrest was confirmed by light microscopy, by staining a small sample of the culture with DAPI followed by fluorescence microscopy, and for a few samples also by flow cytometry. Cells were then washed and incubated in nocodazole-containing YP medium supplemented with 2% galactose to induce expression for 3 hr, after which cells were imaged with a Zeiss Axioplan2 microscope coupled to a Zeiss AxioCam HRC camera. The relative amount of nuclear Whi5-GFP was determined by determining the increase of the intensity of the nuclear Whi5-GFP signal over the Whi5-GFP intensity of the cytoplasm.

### Venn diagrams

Venn diagrams were calculated and visualized using http://bioinformatics.psb.ugent.be/webtools/Venn/.

### Data availability

The authors state that all data necessary for confirming the conclusions presented in the article are represented fully within the article.

## Results and Discussion

### Mapping the SDL network and comparison with previous genetic screens

**Figure 1:**
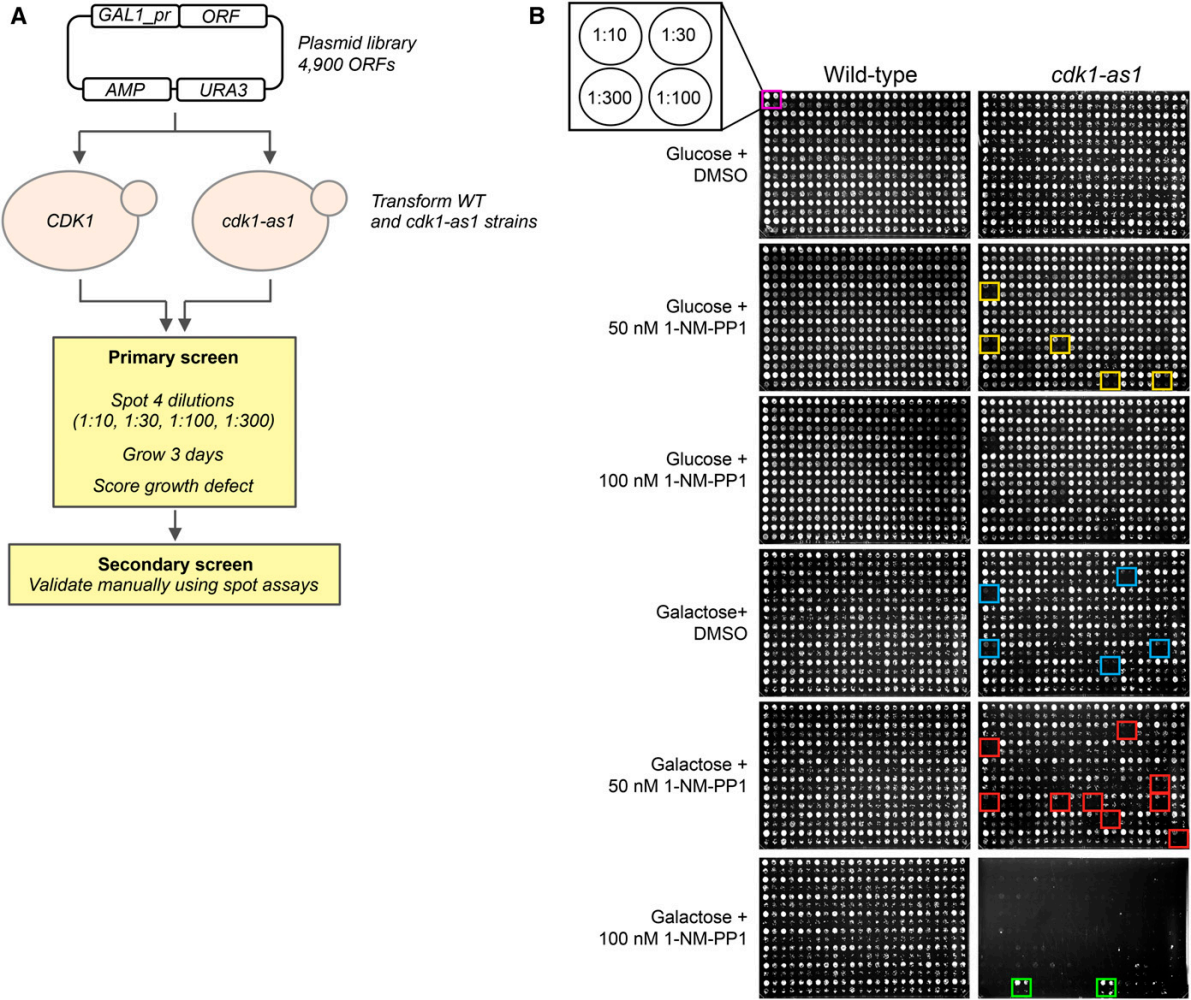
Set-up of the screen. (A) Schematic overview of the screen. (B) Example of the data. Red boxes indicate genes that aggravate the *cdc28-as1* phenotype, whereas green boxes indicate genes that ameliorate the slow-growth defect of *cdc28-as1* mutants on 1-NM-PP1. Yellow boxes indicate genes that induce the SDL phenotype even under noninducing conditions, whereas blue boxes indicate genes that induce the SDL phenotype even in the absence of 1-NM-PP1. DMSO, dimethyl sulfoxide; ORF, open reading frame; SDL, synthetic dosage lethality; WT, wild-type.

We systematically transformed a WT strain and a *cdc28-as1* mutant strain (Bishop *et al.* 2000; Zimmermann *et al.* 2011) with the yeast movable ORF library, which is an arrayed plasmid library consisting of ∼4900 ORFs under control of the galactose-inducible *GAL1* promoter (Gelperin *et al.* 2005). We screened for genes that, when overexpressed, either specifically inhibited the growth of *cdc28-as1* mutant cells (see Figure 1A and *Materials and Methods*) or that (at least partially) complemented the growth defect of *cdc28-as1* mutant cells. We found that overexpression of 366 genes in total resulted in reduced cell viability of the *cdc28-as1* mutant (Figure 1B, red boxes and Supplemental Material, Table S1) and that 20 genes may suppress the growth defect of this mutant (Figure 1B, green boxes). Most of the 366 SDL genes already caused significant growth defects under noninducing conditions (*e.g.*, see Figure 1B, yellow boxes), which is likely due to leakage from the *GAL1* promoter. Furthermore, several SDL genes caused growth defects in the absence of 1-NM-PP-1 (*e.g.*, blue boxes in Figure 1B), which is probably caused by the fact that the *cdc28-as1* allele is hypomorphic (Bishop *et al.* 2000). This indicates that just a slight increase in the expression of these genes is sufficient to perturb cell homeostasis in cells with reduced Cdc28 activity.

**Figure 2:**
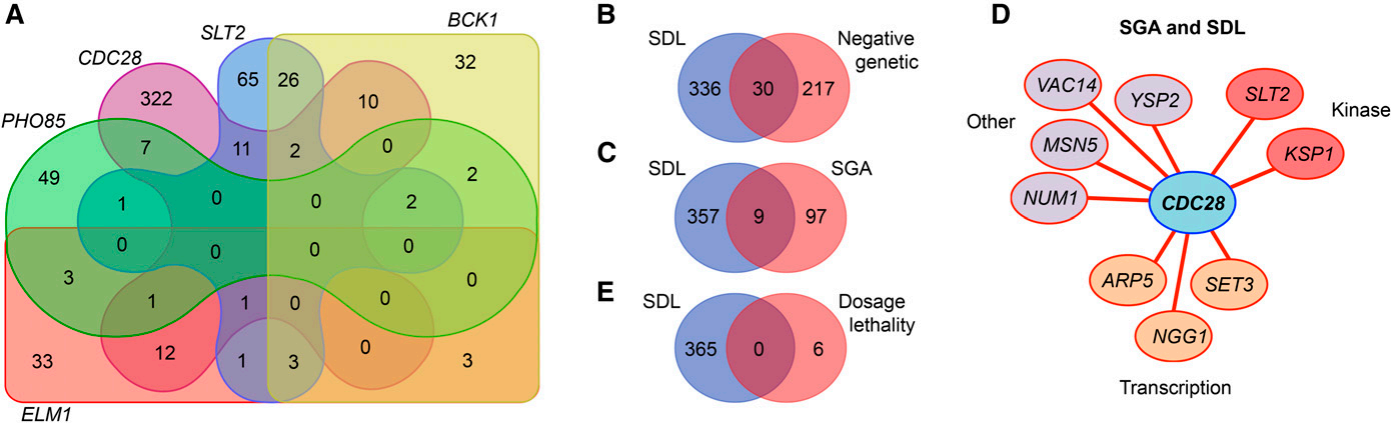
Comparison of the SDL dataset with other genome-wide screens. (A) Overlap between the “hub” SDL networks of *CDC28* and the kinases *SLT2*, *BCK1*, *ELM1*, and *PHO85*. Data were obtained from Sharifpoor *et al.* (2012). (B) Overlap between the SDL screen and previously identified genes with a negative genetic relationship with *CDC28*. Data were obtained from BioGRID. (C and D) Overlap between the SDL screen and the SGA screen described in Zimmermann *et al.* (2011). (E) Overlap between the SDL screen and previously identified genes that cause dosage lethality of *cdc28* mutants (data from BioGRID). SDL, synthetic dosage lethality; SGA, synthetic genetic array.

Notably, the SDL network of *CDC28* is substantially larger than previously identified kinase SDL networks [366 unique SDL interactions for *CDC28* compared to 65 for *PHO85* (Sharifpoor *et al.* 2012)]. This may reflect the importance of Cdc28 in cell cycle control, cell morphogenesis, and cell homeostasis, and is consistent with the previous finding that kinases that coordinate cell cycle progression with cell polarity tend to exhibit “hub” SDL profiles (Sharifpoor *et al.* 2012). Perhaps surprisingly, there appeared to be limited overlap between these kinase SDL hubs (Figure 2A). The reason for the lack of overlap is not clear, but it could reflect the unique cellular functions of these kinases. For example, several of the hub kinases have important functions in response to cellular stress, such as Slt2, which promotes cell survival in response to cell wall stress. In contrast, Cdc28 is important for the regulation of multiple cellular processes under normal growth conditions. An alternative, technical explanation for the relative lack of overlap between these kinase hubs could be that different *GAL* overexpression systems were used with different epitope tags (such as N-terminal GST compared to the C-terminal tag used here).

### Limited overlap between the SDL network and previously identified negative genetic interactions with CDC28

To gain further understanding of the genetic network of *CDC28*, we compared our dataset to genes previously shown to negatively genetically interact with *CDC28*. It has been observed that SGA and SDL screens tend to uncover nonoverlapping genetic interactions, reflecting the distinct properties associated with loss-of-function mutation (SGA) and potential gain-of-function (gene overexpression; SDL) (Measday *et al.* 2005). Indeed, there exists <10% overlap (30 genes) between the SDL dataset and all previously reported negative genetic interactions that were identified with various hypomorphic *cdc28* alleles (Figure 2B and Table S2; see *Materials and Methods*). However, these *cdc28* alleles have different cell cycle defects (Reed and Wittenberg 1990; Reed 1980; Hadwiger *et al.* 1989b; Surana *et al.* 1991). Therefore, to be able to better compare the SDL data with the negative genetic interaction network of *CDC28*, we focused on data from our own previously published SGA screen in which we used the same *cdc28-as1* query mutant as in the current SDL screen (Zimmermann *et al.* 2011). This comparison identified nine genes that were present in both the SDL and the SGA datasets, *i.e.*, *ARP5*, *KSP1*, *MSN5*, *NGG1*, *NUM1*, *SET3*, *SLT2*, *VAC14*, and *YSP2* (Figure 2, C and D and Table S3). The fact that these genes display both a synthetic lethal as well as a dosage lethal interaction with *CDC28* shows that cells are very sensitive to changes in the activity of these genes when Cdc28 activity has been compromised, suggesting that these genes and Cdc28 have similar cellular functions. Supporting this interpretation, three out of the nine genes are involved in the regulation of basal transcription (*i.e.*, *ARP5*, *NGG1*, and *SET3*), and we have recently shown that Cdc28 promotes basal transcription of housekeeping genes to maintain cell homeostasis (Chymkowitch *et al.* 2012).

### Genes that aggravate the cdc28-as1 slow growth phenotype

According to information retrieved from the SGD database, only six genes have previously been shown to induce dosage lethality in *cdc28* mutants, *i.e.*, *CLN1* (Tobe *et al.* 2009), *ELM1* (Sharifpoor *et al.* 2012), *FCP1* (Chymkowitch *et al.* 2012), *MPS1* (Rudner *et al.* 2000), *RTR1* (Chymkowitch *et al.* 2012), and *SSU72* (Chymkowitch *et al.* 2012). Surprisingly, the SDL screen did not identify any of these six genes (Figure 2E), even though three of these interactions were identified previously by our own laboratory using the same *cdc28-as1* mutant [*i.e.*, *RTR1*, *SSU72*, and *FCP1* (Chymkowitch *et al.* 2012)]. This finding reflects the previous observation that SDL screens can result in a false negative rate as high as 50% (Sharifpoor *et al.* 2012). There may be multiple explanations for this high false negative rate. For instance, not all ORFs are present in the SDL library, such as *e.g.*, *YKL048C/ELM1*. Furthermore, we have not fully sequenced the plasmids harboring these ORFs and it is possible that at least some of them either do not contain the correct ORF, or they may contain loss-of-function mutations resulting from the PCR step during library construction. Furthermore, overexpression of highly toxic genes may result in rapid selection of cells that have acquired mutations in either their genetic background or in the plasmid, resulting in bypass of the SDL phenotype. It is also possible that the activity of the overexpressed proteins is impaired by the relatively large C-terminal ProtA-HA-HIS_6_ tag, thus suppressing the SDL phenotype. Keeping in mind these drawbacks, which are inherent to high-throughput screens, we believe that the SDL dataset provides useful information about the function of Cdc28.

**Figure 3:**
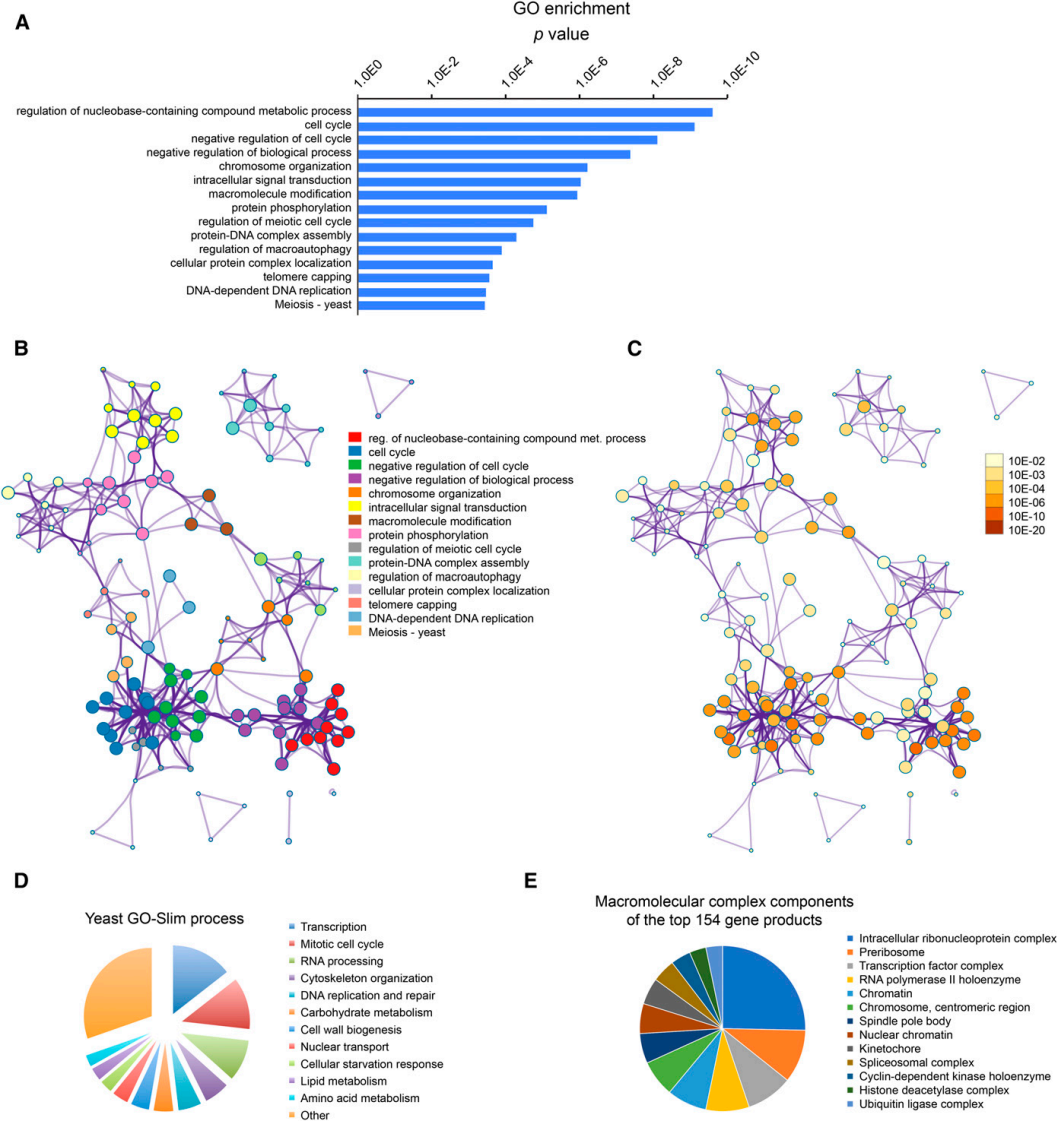
Analysis of the data reveals overrepresentation of genes involved in the cell cycle, DNA metabolism, and transcription. (A) GO Biological Process analysis of the genes that aggravate the *cdc28-as1* phenotype (negative SDL interactions). GO analysis was performed using Metascape. (B) Network plot of the relationships among GO terms. Nodes represent enriched terms colored by its cluster ID. (C) The same network as presented in (B) but showing *P* values for the nodes. (D) Analysis of the negative SDL interactors using the GO Slim mapper tool of the SGD. (E) Analysis of the macromolecular complex components of the top 154 genes in the SDL network. Only those GO terms are shown for which at least five terms genes were identified. GO, gene ontology; ID, identifier; SDL, synthetic dosage lethality; SGD, *Saccharomyces* Genome Database.

To better understand the SDL network, we analyzed the functions of the genes in the SDL dataset. GO analysis revealed overrepresentation of genes important for the cell cycle, nucleic acid metabolism, and transcription (Figure 3, A–E), as might be expected based on the important functions of Cdc28 in these processes (Enserink and Kolodner 2010). STRING analysis of the relationships between the proteins encoded by these genes yielded an interaction network centered on Cdc28 (Figure S1, A and B), which was highly enriched for factors involved in cell cycle control (1.58E−08; Figure S1C). Thus, as previously reported (Sharifpoor *et al.* 2012), SDL screening is a powerful tool for functional characterization of kinases, and while we cannot discuss all the SDL genes in detail, we will highlight some of the major overrepresented groups below.

### Cell cycle regulators

**Figure 4:**
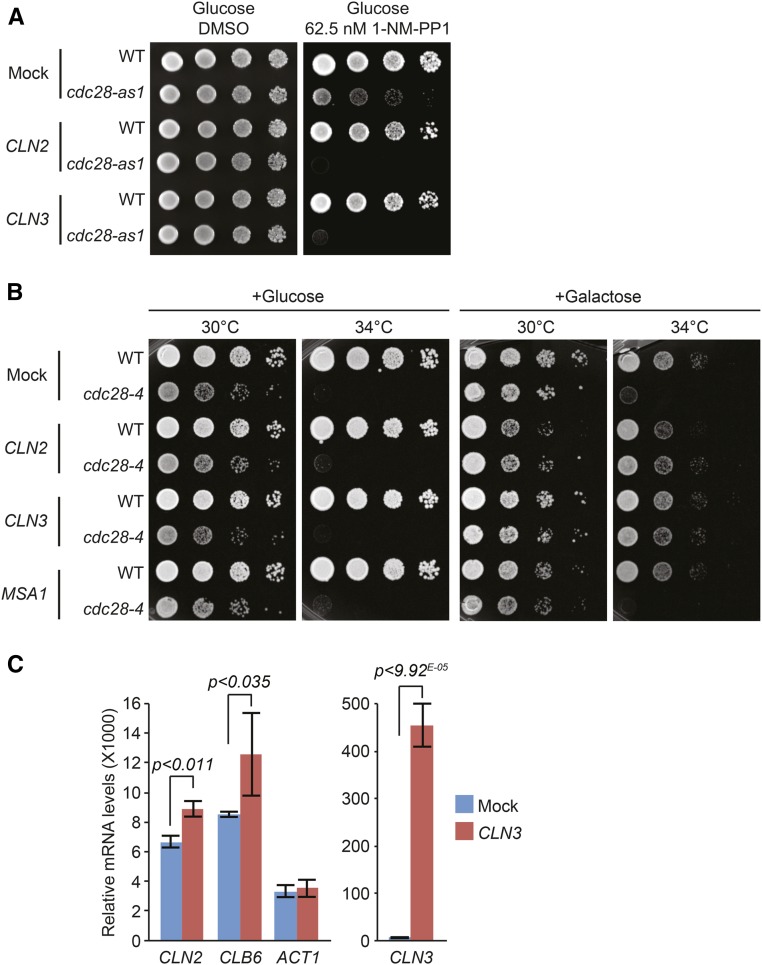
The effect of overexpression of cyclins. (A) Overexpression of *CLN2* and *CLN3* causes SDL in *cdc28-as1* mutants even under noninducing conditions. Cultures of cells transformed with plasmids containing the ORFs *YEL074W* (“Mock”), *CLN2*, or *CLN3* were spotted on glucose-containing SC-URA plates supplemented with either DMSO or 1-NM-PP1, followed by incubation at 30° until colonies appeared. (B) Overexpression of *CLN2* and *CLN3* suppresses the growth defect of *cdc28-4* mutants. Cultures of cells transformed with plasmids containing the control ORF *YEL074W* (“Mock”), *CLN2*, *CLN3*, or *MSA1* were spotted on YPD or on YP-galactose and incubated at either 30° or 34° until colonies appeared. (C) *cdc28-as1* strains transformed with plasmids harboring either the control ORF *YEL074W* (Mock) or *CLN3* were grown to log phase and treated with 500 nM 1-NM-PP1, after which transcription was induced with galactose as described in the *Materials and* *Methods*. Subsequently, mRNA levels of *CLN2*, *CLB6*, *ACT1*, and *CLN3* were determined by RT-qPCR. DMSO, dimethyl sulfoxide; ORF, open reading frame; RT-qPCR, quantitative reverse transcription polymerase chain reaction; SC-URA, synthetic complete-uracil; SDL, synthetic dosage lethality; WT, wild-type; YP, yeast extract peptone; YPD, YP and dextrose.

*CLN2* and *CLN3*: we found that increased expression of multiple cell cycle genes resulted in impaired survival of *cdc28-as1* mutants. In particular, *cdc28-as1* mutants were very sensitive to overexpression of *CLN2* and *CLN3*, which caused SDL in the absence of galactose (*i.e.*, noninducing conditions; Figure 4A), indicating that even a minor increase in the levels of these cyclins (due to leakage of the *GAL1* promoter) is toxic to these cells. This was surprising because previous studies had identified cyclins as high dosage suppressors of temperature-sensitive *cdc28* alleles; for instance, *CLN1* and *CLN2* suppress *cdc28-4*, *cdc28-9*, and *cdc28-13*, whereas B-type cyclins suppress *cdc28-1N* mutants (Hadwiger *et al.* 1989b; Surana *et al.* 1991). This incongruity between the different *cdc28* mutants could be due to the fact that the *cdc28-as1* allele confers a different cell cycle defect than the aforementioned temperature-sensitive *cdc28* alleles; for instance, *cdc28-4* mutant cells primarily have a defect early in the cell cycle, whereas *cdc28-as1* mutants mainly have a M phase defect (Reed 1980; Hadwiger *et al.* 1989b; Surana *et al.* 1991; Bishop *et al.* 2000). Consistent with this idea, we found that overexpression of *CLN2* and *CLN3* in the *cdc28-4* mutant suppressed the temperature-sensitive growth defect, whereas overexpression of the control ORFs *YEL074W* and *MSA1* had no effect (Figure 4B).

One explanation for the toxicity of overexpressed cyclins in *cdc28-as1* mutant cells could be that these cells spend much more time in the M phase of the cell cycle when exposed to sublethal doses of 1-NM-PP1 (Zimmermann *et al.* 2011), and that overexpression of *CLN2* or *CLN3* in this phase of the cell cycle titrates away B-type cyclins and inappropriately triggers activation of the G1/S phase program. We tested this by overexpressing *CLN3* in the *cdc28-as1* mutant strain treated with 500 nM 1-NM-PP1. This dose of drug has been reported to strongly delay cells in early M phase (Bishop *et al.* 2000), which we confirmed by flow cytometry (Figure S2A). We then analyzed activation of the G1 transcriptional program by monitoring the transcription of *CLN2* and *CLB6*, which are part of the G1 cluster of cell cycle-regulated genes (Spellman *et al.* 1998). Interestingly, overexpression of *CLN3* in these M phase-arrested mutants resulted in a modest but significant increase in *CLN2* and *CLB6* mRNA levels, whereas expression of the actin-encoding gene *ACT1* remained unchanged (Figure 4C). In contrast, overexpression of *CLN3* in M phase-arrested *cdc28-4* mutants did not result in increased expression of *CLN2* and *CLB6* (Figure S2B). This indicates that overexpression of *CLN3* can to a certain degree activate the G1/S phase transcriptional program specifically in *cdc28-as1* cells. This supports our hypothesis that ectopic expression of *CLN3* in M phase-arrested *cdc28-as1* mutants may inappropriately activate cellular processes normally associated with early stages of the cell cycle, thus contributing to the SDL phenotype. In addition, overexpressed Cln3 may further exacerbate the SDL phenotype by outcompeting the binding of Clb1-4 to Cdc28, resulting in reduced Clb-Cdc28 activity.

We identified several other cell cycle regulators in the SDL screen, which we will only discuss briefly. 

*SIC1*: Sic1 is an inhibitor of Clb5/6–Cdc28 complexes (Schwob *et al.* 1994), and phosphorylation of Sic1 by Cdc28 promotes cell cycle entry. Depending on the cell cycle stage, *SIC1* overexpression can result in cell cycle arrest (Nugroho and Mendenhall 1994), but it can also prematurely induce the formation of prereplicative complexes at origins of replication, causing some cells to rereplicate their DNA (Dahmann *et al.* 1995). Overexpression of *SIC1* may be toxic to the hypomorphic *cdc28**-as1* mutant by preventing efficient cell cycle progression, by interfering with the timing of DNA replication, or both. 

*CDC20*: Cdc20 is an activator of the anaphase promoting complex (Visintin *et al.* 1997). Mitotic Cdc28 activity is required to activate Cdc20, and *cdc28* mutants have difficulty leaving mitosis (Rudner and Murray 2000). Overexpression of Cdc20 under conditions of reduced Cdc28 activity likely interferes with regulation of mitosis, potentially inducing premature entry or exit from mitosis leading to genome instability.

### DNA replication

*CDC6*, *ORC2*, *SLD2*, *POL12*, *POL32*, *RFA1*, *TOF1*, and *TOP2*: Sld2, Orc2, and Cdc6 are important for initiation of DNA replication (Kamimura *et al.* 1998) (Bell and Stillman 1992; Hartwell 1976; Park and Sternglanz 1999); Rfa1 is a component of the heterotrimeric replication protein A complex, which is essential for DNA replication (Brill and Stillman 1991); and Tof1 is a component of the DNA replication complex and is particularly important for recovery from DNA replication stress (Park and Sternglanz 1999; Katou *et al.* 2003). Pol12 is a component of the DNA polymerase α-primase complex which is required for initiation of DNA replication (Foiani *et al.* 1994), whereas Pol32 is a component of DNA polymerase δ and is involved in efficient DNA replication (Gerik *et al.* 1998). Top2 is the catalytic subunit of topoisomerase II (Holm *et al.* 1985), and plays a major role in maintaining genome stability by alleviating DNA supercoiling and catenation that arise as a consequence of DNA replication. Changes in the expression levels of *RFA1*, *ORC2*, *CDC6*, *SLD2*, *POL32*, *TOF1*, and *TOP1* may either perturb the stoichiometry of the factors involved in DNA replication, disturb the timing of DNA replication, or affect DNA supercoiling. For instance, overexpression of fission yeast *CDC6* is sufficient to trigger DNA replication (Nishitani and Nurse 1995). We and others have previously shown that minor changes in the timing and efficiency of DNA replication can have severe consequences for the viability of cells with compromised Cdc28 activity (Tanaka and Diffley 2002; Enserink *et al.* 2009; Lengronne and Schwob 2002). For instance, Cdc28 activity is required for the repair of DNA double-strand breaks (Ira *et al.* 2004; Aylon *et al.* 2004), and even a slight reduction in Cdc28 activity can result in mitotic catastrophe and loss of cell viability in response to DNA damage emanating from problems with DNA replication (Enserink *et al.* 2009). However, there may exist additional mechanisms by which overexpression of these genes causes SDL of *cdc28-as1* mutants; overexpression of *CDC6* from the *GAL1* promoter is known to cause an M phase delay (Bueno and Russell 1992), and because Cdc28 activity is required for entry into M phase (Pines and Hunter 1990) it is possible that a combination of high Cdc6 levels with reduced Cdc28 activity results in M phase arrest.

### Chromosome cohesion

*SCC1/MCD1*: Chromosome cohesion occurs concomitantly with DNA replication (Uhlmann and Nasmyth 1998). The α-kleisin subunit Mcd1 is involved in recruiting the cohesion complex to chromosomes (Guacci *et al.* 1997; Michaelis *et al.* 1997). DNA replication-induced chromosome cohesion is limited to S phase, and this is regulated by Cdc28. Specifically, Cdc28 inhibits chromosome cohesion after S phase (Lyons and Morgan 2011). Thus, overexpression of cohesion factors in a cell with reduced Cdc28 activity may result in establishment of cohesion outside of S phase, leading to chromosome separation defects in anaphase.

### Chromosome condensation

*SMC4*: After DNA replication, chromosomes are compacted by the Smc2/4 condensin complex. Chromosome condensation is regulated by Cdc28 (Enserink and Kolodner 2010), and Smc4 is activated by Cdc28 in this process (Robellet *et al.* 2015). In fact, Smc4 is highly sensitive to Cdc28 activity, such that initiation of chromosome condensation is induced at levels of Cdc28 activity that are too low to activate other mitotic processes (Robellet *et al.* 2015). Therefore, one can envision that overexpression of *SMC4* in a *cdc28-as1* mutant results in high levels of unphosphorylated Smc4, such that the cell fails to reach the critical level of Smc4 phosphorylation required for inducing chromosome condensation.

### Mitotic spindle, kinetochore, and spindle pole body

**Figure 5:**
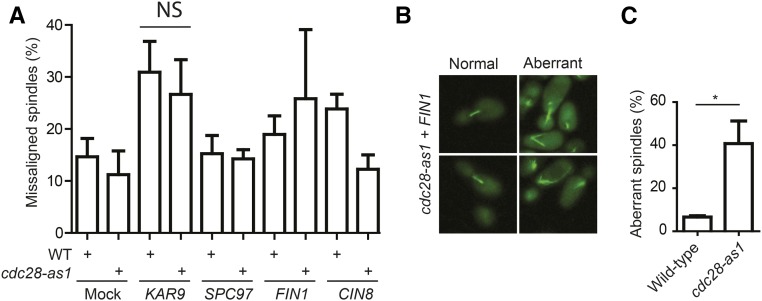
Overexpression of *FIN1* in *cdc28-as1* mutants induces mitotic spindle aberrancies. (A) No obvious differences in spindle misalignment between WT cells and *cdc28-as1* mutants after overexpression of *KAR9*, *SPC97*, *FIN1*, or *CIN8*. *TUB1-GFP*-expressing WT cells and *cdc28-as1* mutants were transformed with plasmids containing the indicated ORFs (Mock: *YEL074W*), after which cells were treated with 1-NM-PP1 and overexpression was induced by galactose as described in *Materials and Methods*. Spindle alignment in at least 100 M phase cells was imaged and quantified using fluorescence microscopy. (B) Overexpression of *FIN1* in the *cdc28-as1* mutant background results in aberrant spindle assembly. Cells were treated and imaged as in (A). At least 300 cells were analyzed per treatment and genotype. (C) Quantification of the data shown in (B). Error bars indicate SD. *P < 0.05. NS, not significant; WT, wild-type.

*CIN8*, *CSE4*, *SGO1*, *FIN1*, *KAR9*, *MAD3*, *SLK19*, *MPS2*, *SPC29*, and *SPC97*: Cin8 is a kinesin motor protein involved in assembly of the mitotic spindle (Saunders and Hoyt 1992); Cse4 is a histone H3-like protein that is a component of the kinetochore (Stoler *et al.* 1995), whereas Sgo1 is a kinetochore protein that senses tension on the mitotic spindle (Indjeian *et al.* 2005); Fin1 is an intermediate filament protein that forms filaments between spindle pole bodies, which is important for mitotic spindle stability (van Hemert *et al.* 2002); Kar9 is involved in spindle positioning (Miller and Rose 1998); and Mad3 is a component of the mitotic spindle checkpoint. Formation of the mitotic spindle is strictly regulated by Cdc28 (Enserink and Kolodner 2010). Therefore, overexpression of these proteins under conditions of reduced Cdc28 activity could perturb spindle assembly, potentially resulting in chromosome missegregation and cell death. To test this, we overexpressed *KAR9*, *SPC97*, *FIN1*, and *CIN8*, and monitored mitotic spindle assembly and alignment in strains expressing Tub1-GFP (Figure 5A and Figure S2C). Consistent with the function of Kar9 in spindle positioning under normal conditions (Miller and Rose 1998), we found that overexpression of *KAR9* resulted in an increase in the number of cells with misaligned spindles, although there was no difference between WT and *cdc28-as1* cells (Figure 5A). Furthermore, and consistent with the previous observation that overexpression of *KAR9* induces migration of the nucleus into the bud (Korinek *et al.* 2000), we found that *KAR9* overexpression resulted in increased translocation of the entire mitotic spindle into the bud (Figure S2, D and E). Together, these data indicate that the SDL phenotype of *KAR9* overexpression may be caused by increased misalignment or mislocalization of the mitotic spindle, but we cannot exclude the possibility that Cdc28 activity is also required for the survival of cells with such misaligned/mislocalized spindles.

While spindle alignment did not appear to be affected in response to overexpression of *SPC97*, *FIN1*, and *CIN8*, we did observe that overexpression of *FIN1* resulted in substantial malformation of the mitotic spindle in ∼40% of the *cdc28-as1* cells, and these spindles often had unusually long astral microtubules (Figure 5, B and C). This phenotype is remarkably similar to that of cells overexpressing *fin1-5A*, which encodes a form of Fin1 that can no longer be phosphorylated by Cdc28 (Woodbury and Morgan 2007). Therefore, the SDL phenotype is likely caused by accumulation of unphosphorylated Fin1. Currently, we do not know what causes the SDL phenotype imparted by overexpression of *SPC97* and *CIN8*, which did not cause obvious spindle defects in either the WT cells or in *cdc28-as1* mutants (Figure 5A and Figure S2C). It is possible that overexpression of these genes results in phenotypes too subtle to be detected in our assays. For instance, overexpression of *CIN8* has been shown to induce premature spindle elongation in WT cells, but it was only clearly detectable when cells were maintained in prolonged hydroxyurea-induced S phase arrest (Saunders *et al.* 1997).

### Cell morphogenesis

*BEM1*, *BUD2*, and *BUD4*: Cell cycle entry is accompanied by the assembly of a new bud (Schwann 1837), which is driven by cell polarity factors and which strongly depends on the cell cycle (Howell and Lew 2012). Bem1 is an adaptor protein that, together with the small GTPase Bud1, is important for bud site selection and bud growth (Bender and Pringle 1991). Bud2 is a GTPase-activating protein (GAP) for Bud1 (Benton *et al.* 1993; Cvrckova and Nasmyth 1993), whereas the anillin-like protein Bud4 is important for formation of the septin ring (Eluere *et al.* 2012), which is an essential aspect for bud assembly. Bud growth is dependent on Cdc28 activity (Hartwell *et al.* 1973; McCusker *et al.* 2007; Howell and Lew 2012), Bem1 and Bud4 are direct Cdc28 targets (Han *et al.* 2005; Eluere *et al.* 2012), and it is likely that altered levels of Bem1, Bud2, and Bud4 perturb bud growth under conditions of reduced Cdc28 activity. Bem1 may also affect cell cycle progression, since *BEM1* overexpression has previously been shown to enhance G1 arrest (Lyons *et al.* 1996) in response to α factor. Because α factor inhibits Cdc28 activity (Tyers and Futcher 1993), it is conceivable that overexpression of *BEM1* in the hypomorphic *cdc28-as1* mutant background results in G1 phase arrest.

### Transcription

A very large number of genes that induce dosage lethality in the *cdc28-as1* mutant are involved in transcription. This is not surprising, given the fact that Cdc28 is important for activation of transcription at several stages (Chymkowitch and Enserink 2013). For instance, Cdc28 regulates a compendium of cell cycle-specific transcription factors that play an important role in executing the various stages of the cell cycle (Enserink and Kolodner 2010; Wittenberg and Reed 2005). Cdc28 also directly controls the basal transcription machinery at a subset of genes mainly involved in housekeeping and cell homeostasis (Chymkowitch *et al.* 2012; Chymkowitch and Enserink 2013). Consistently, analysis by GO Slim Mapper revealed that nearly all of the SDL-inducing genes involved in the process of transcription are part of complexes that regulate basal transcription, such as TFIID, INO80, Mediator, and the HDAC and HAT complexes, as well as the RNA polymerase II holoenzyme (Table S4). It is likely that overexpression of these genes alters the stoichiometry of these complexes, thereby interfering with efficient transcription. Consistently, we have previously shown that transcription of cell cycle genes (such as cyclins) is strongly affected by even very modest changes in transcription efficiency (Zimmermann *et al.* 2011), which is likely to have severe consequences for the cell cycle progression and viability of 1-NM-PP1-treated *cdc28-as1* cells.

### Genes that may alleviate the cdc28-as1 slow growth phenotype

**Figure 6:**
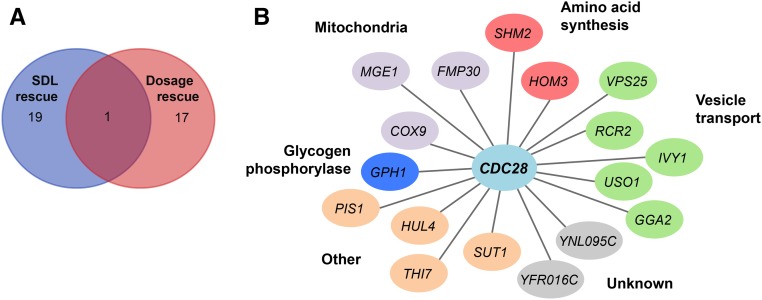
Genes that may promote dosage rescue of *cdc28-as1* mutant cells. (A) Comparison of previously identified genes that mediate dosage suppression of *cdc28* alleles with the ORFs that mediate dosage suppression identified by the synthetic dosage lethality (SDL) screen. The only ORF in common between these datasets was *YBR160W*, which encodes Cdc28. (B) Putative dosage suppressors identified by the SDL screen organized by their cellular functions.

We identified 20 potential suppressor genes that may partially compensate the growth defect of the *cdc28-as1* mutant (Table S1). We compared these genes to previously identified dosage suppressors of *cdc28* alleles (Figure 6A) and found that, apart from *CDC28* itself, there was no overlap between the SDL screen and these previous dosage rescue studies, which as discussed above mainly identified cyclins. GO analysis did not reveal any overrepresented gene functions of the genes that alleviate the *cdc28-as1* growth defect. However, we did notice that five of these genes (*GGA2*, *VPS25*, *RCR2*, *IVY1*, and *USO1*) have functions in vesicle transport (Nakajima *et al.* 1991; Zhdankina *et al.* 2001; Robinson *et al.* 1988; Kota *et al.* 2007; Lazar *et al.* 2002) (Figure 6B), indicating that one consequence of chronically reduced Cdc28 activity could be defective vesicle transport, which might be overcome by overexpression of these vesicle transport genes. Indeed, Cdc28 has been shown to be important for membrane trafficking (McCusker *et al.* 2012).

In addition to genes involved in vesicle transport, we identified genes involved in amino acid synthesis. We have previously shown that deletion of one of these genes, *HOM3* (which encodes aspartokinase), results in synthetic lethality with the *cdc28-as1* mutation (Zimmermann *et al.* 2011). Thus, loss of *HOM3* aggravates the growth defect of *cdc28-as1* mutants whereas its overexpression suppresses this defect, suggesting that *HOM3* may have an important function in the cell cycle. Indeed, we previously found that *HOM3* may support survival of the *cdc28-as1* mutant cells by promoting the synthesis of dNTPs (Zimmermann *et al.* 2011).

Another gene worth mentioning is *GPH1*. Gph1 functions as glycogen phosphorylase, which mobilizes glycogen to increase glucose-1-phosphate. Intriguingly, Gph1 was recently found to be a direct Cdc28 substrate and phosphorylation of Gph1 may coordinate carbohydrate metabolism with cell division (Zhao *et al.* 2016), providing a potential explanation for why its overexpression suppresses the *cdc28-as1* phenotype. Interestingly, in addition to Gph1, the SDL screen identified several Cdc28 targets (see below), although all of them (except Gph1) induced a dosage lethality phenotype.

### Overrepresentation of Cdc28 targets

It was previously reported that SDL screens can identify novel functional relationships and pathways regulated by kinases, and SDL screens are enriched for kinase substrates (Sharifpoor *et al.* 2012; Sopko *et al.* 2006). Interestingly, our SDL screen was also enriched for direct targets of Cdc28 (*Tab2* in Table S1). We identified 29 of the 100 currently known Cdc28 targets (known Cdk1 targets are defined here as proteins shown in low-throughput experiments to be phosphorylated in a Cdk1-dependent manner *in vivo*; we previously compiled a list of targets (Enserink and Kolodner 2010) to which we added more recently identified Cdk1 substrates). However, given that 20 known Cdc28 targets were not present in the library and that at least three clones were incorrect, we identified 37% of the currently known Cdc28 substrates that could theoretically be identified by the SDL screen.

To gain further insight in potential Cdc28 targets, we compared the SDL dataset to previously published datasets. We compiled data obtained with phosphoproteomics, physical interactions, and *in vitro* kinase assays (see *Materials and Methods*). Figure S3A shows a Venn diagram that combines all these datasets, including a list of known Cdc28 targets (see Table S5 for ORF identities). This comparison indicates that only a minority of direct Cdc28 substrates has been found to actually physically interact with the Cdc28 holoenzyme (Figure S3B and Table S6), suggesting that phosphorylation by Cdc28 involves transient interactions of the Cdc28 holoenzyme with its substrates that elude detection by high-throughput proteomic approaches.

**Figure 7:**
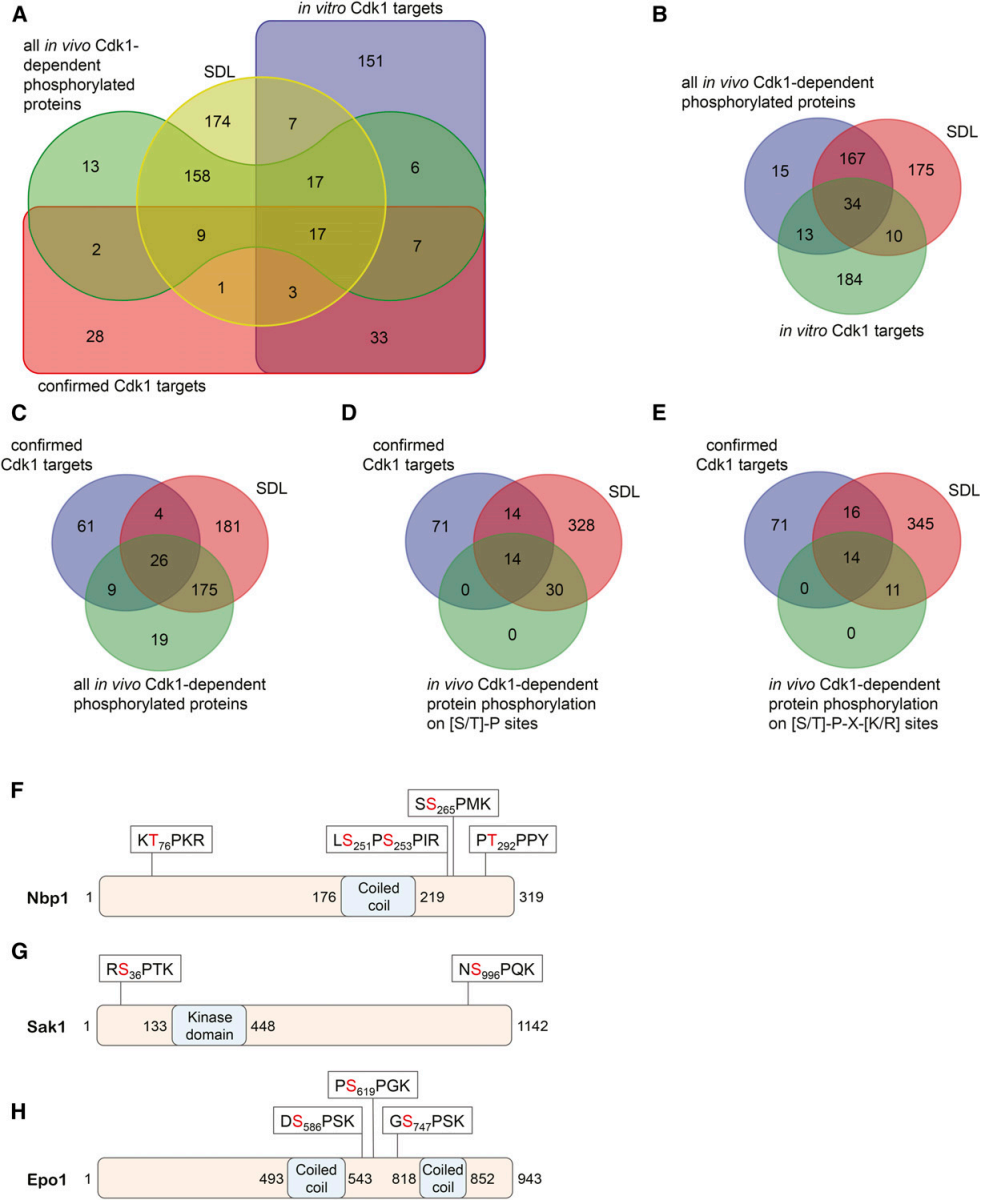
The SDL dataset is enriched for proteins that are phosphorylated in a Cdc28-dependent manner. (A) Venn diagram depicting overlap between the SDL dataset and proteins that are phosphorylated in a Cdc28-dependent manner *in vivo* and *in vitro*, and which have previously been confirmed to be Cdc28 substrates. (B) Most proteins that have been found to be phosphorylated in a Cdc28-dependent manner *in vivo* cause dosage lethality when overexpressed in a *cdc28-as1* mutant. The Venn diagram shows overlap between the SDL dataset and proteins that are phosphorylated in a Cdc28-dependent manner *in vitro* and *in vivo*. (C) The SDL dataset is enriched for known Cdc28 targets. The Venn diagram shows overlap between the SDL screen, proteins that have been found to be phosphorylated in a Cdc28-dependent manner *in vivo*, and previously confirmed Cdc28 substrates. (D and E) Overexpression of proteins phosphorylated on potential Cdc28 sites cause an SDL phenotype. The Venn diagrams show overlap between the SDL dataset, confirmed Cdc28 targets, and proteins that are known to be phosphorylated *in vivo* on either minimal Cdc28 sites (D) or on full Cdc28 consensus sites (E) in a Cdc28-dependent manner. (F–H) Domain structures of Nbp1 (F), Sak1 (G), and Epo1 (H), and location of sites known to be phosphorylated *in vivo* in a Cdc28-dependent manner. CDK, cyclin-dependent kinase; SDL, synthetic dosage lethality.

To simplify the interpretation of the data, we removed the physical interaction dataset from the Venn diagram (Figure 7A and Table S7), as well as the dataset containing known Cdc28 targets (Figure 7B and Table S8). This revealed that most (201 out of 229) proteins that have previously been found to be phosphorylated *in vivo* in a Cdc28-dependent manner also cause dosage lethality of *cdc28-as1* mutants, suggesting a functional relationship between these proteins and Cdc28. However, it is unlikely that many of these proteins are direct Cdc28 targets, because most of them do not appear to be phosphorylated by Cdc28 *in vitro* (Figure 7B and Table S8). Furthermore, even though the phosphorylation of these proteins depends on Cdc28 activity, a large number of these phosphorylations occur on non-Cdc28 consensus sites [see Datasets S1 and S2 in Holt *et al.* (2009)]. While Cdc28 has been shown to be able to phosphorylate substrates on nonconsensus sites (Harvey *et al.* 2005; McCusker *et al.* 2007), this appears to be relatively rare (Suzuki *et al.* 2015). Therefore, it is more likely that Cdc28-dependent phosphorylation of proteins on nonconsensus sites in the SDL dataset depends on cell cycle stage rather than on Cdc28 activity *per se*. Alternatively, Cdc28 may indirectly control their phosphorylation by regulating kinases/phosphatases that in turn regulate phosphorylation of these proteins. It should also be mentioned that high-throughput phosphoproteomics studies that used the hypomorphic *cdc28-as1* allele may have missed poor substrates whose phosphorylation is very sensitive to Cdk1 activity; as a consequence, these sensitive substrates are absent from our comparisons.

We also compared the SDL dataset with the list of all known Cdc28 targets, as well as with the list of proteins shown to be phosphorylated *in vivo* in a Cdc28-dependent manner. There was substantial overlap between these datasets (Figure 7C and Table S9). To be able to pinpoint potential novel, direct Cdc28 substrates, we then focused only on those proteins that are phosphorylated in a Cdc28-dependent manner *in vivo* on either minimal Cdc28 sites (S/T-P; Figure 7D and Table S10) or optimal Cdc28 consensus sites (S/T-X-K/R; Figure 7E and Table S11). Interestingly, this revealed that all these proteins were also present in the SDL dataset. We believe that it is likely that these proteins are direct Cdc28 substrates. One example of a likely direct Cdc28 target is the spindle pole body component Nbp1 (Figure 7F), because it induces SDL in a *cdc28-as1* background, it is an efficient *in vitro* Cdc28 target (Ubersax *et al.* 2003), it is phosphorylated *in vivo* on optimal Cdc28 consensus sites in a Cdc28-dependent manner (Bodenmiller *et al.* 2010; Holt *et al.* 2009), and it physically interacts with Cdc28 (Ear *et al.* 2013). Nbp1 is essential for bipolar spindle formation, and it is required for the efficient insertion of the spindle pole body into the nuclear membrane. The effect of Nbp1 phosphorylation by Cdc28 will be the subject of future studies.

Another example of a potential Cdc28 target is Sak1 (Figure 7G), a kinase with similarity to mammalian LKB. Sak1 controls the Swi/Snf1 complex and has been found to be phosphorylated on two optimal Cdc28 consensus sites, S_36_PTK and S_966_PQK, in a Clb2–Cdc28-dependent manner, although the functional significance of these phosphorylations has not been established (Holt *et al.* 2009). SWI/Snf has an important function in cell cycle regulation, and conversely its activity is regulated by the cell cycle (Sif *et al.* 1998; Ruijtenberg and van den Heuvel 2015). Thus, one mechanism by which Cdc28 might regulate SWI/Snf1 activity could be through phosphorylation of Sak1.

Finally, Epo1 may also be a direct Cdc28 target (Figure 7H). Epo1 encodes a protein involved in ER-septin tethering by binding the septin Shs1 and the ER protein Scs2, which is important for creating a diffusion barrier between the mother and daughter cell (Chao *et al.* 2014). Epo1 has been found to be phosphorylated on multiple residues in large-scale phosphoproteomic studies, three of which are optimal Cdc28 consensus sites: S_586_PSK, S_619_PGK, and S_747_PSK. The phosphorylation of at least one of these sites, *i.e.*, S586, is dependent upon Cdc28 activity *in vivo* (Holt *et al.* 2009). Interestingly, the interaction between Epo1 and Scs2 is cell cycle-dependent (Neller *et al.* 2015), indicating that Cdc28 may play an important role in the regulation of the tethering of the ER to the vacuole by regulating the interaction between Epo1 and Scs2.

**Figure 8:**
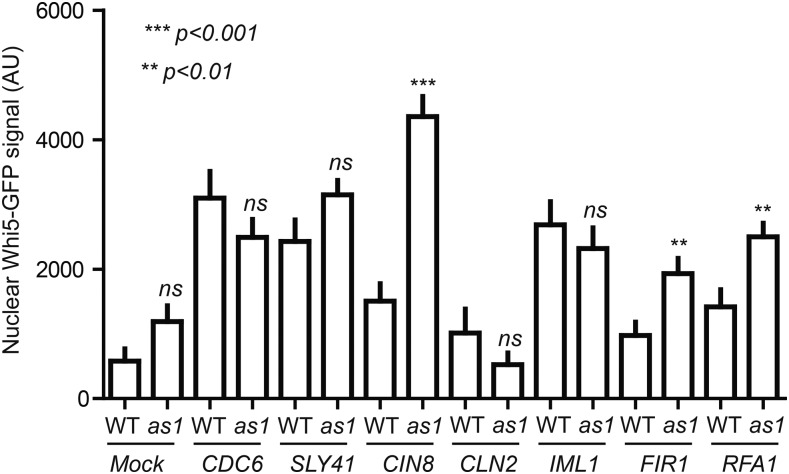
Aberrant nuclear localization of Whi5 in M phase upon overexpression of several SDL genes. Overexpression of *CIN8*, *FIR1*, and *RFA1* results in a significant increase in nuclear localization of Whi5-GFP in M phase cells. Whi5-GFP-expressing cells containing *YEL074W* (“Mock”), *CDC6*, *SLY41*, *CIN8*, *CLN2*, *IML1*, *FIR1*, and *RFA1* expression plasmids were grown to log phase in the presence of glucose and arrested in M phase with nocodazole. Cells were then washed and incubated in nocodazole-containing medium supplemented with galactose to induce expression for 3 hr, after which nuclear localization of Whi5-GFP was assessed by fluorescence microscopy. Error bars indicate SD. ns, not significant; **P < 0.001, ***P < 0.0001. GFP, green fluorescent protein; SDL, synthetic dosage lethality; WT, wild-type.

It is not entirely clear why Cdc28 substrates are enriched in the SDL screen, but one explanation could be that overexpression of these substrates results in competition with other Cdc28 targets for phosphorylation by Cdc28. We tested this hypothesis by monitoring phosphorylation of the direct Cdc28 target Whi5, which is a transcriptional repressor of the G1 transcriptional program (de Bruin *et al.* 2004; Costanzo *et al.* 2004). In late G1 phase, phosphorylation of Whi5 by Cdc28 results in its release from the transcription factors SBF and MBF to accelerate the G1/S transition (Costanzo *et al.* 2004). Phosphorylated Whi5 is transported out of the nucleus and only reenters the nucleus at the end of M phase when Cdc28 is inactivated (Costanzo *et al.* 2004; Taberner *et al.* 2009), and nuclear localization of Whi5-GFP is a highly sensitive read-out for Cdc28 activity (Talia *et al.* 2007; Skotheim *et al.* 2008). We nocodazole-arrested cells in M phase, when Whi5 is hyperphosphorylated by Cdc28 (Taberner *et al.* 2009), and overexpressed the known Cdc28 targets *CDC6*, *SLY41*, *CIN8*, and *FIR1*, as well as *IML1* and *RFA1*, which are not currently known to be Cdc28 targets, and monitored cellular localization of Whi5-GFP (Figure 8; see Figure S4 for examples). We overexpressed the mock ORFs *YEL074W* and *CLN2* as controls; as expected, overexpression of neither of these genes resulted in nuclear localization of Whi5 (Figure 8). However, increased nuclear localization of Whi5-GFP could be observed in the nucleus of cells overexpressing *CDC6*, *SLY41*, and *IML1*, although there was no significant difference between WT and *cdc28-as1* cells (Figure 8). In contrast, overexpression of *CIN8*, *FIR1*, and *RFA1* did result in significantly increased nuclear localization of Whi5-GFP in *cdc28-as1* mutants compared to WT cells (Figure 8). These data indicate that overexpression of certain proteins, including known and potential Cdc28 substrates, can interfere with normal Cdc28 signaling. Thereby, at least some of these proteins may effectively function as Cdc28 inhibitors when overexpressed, providing an explanation for the SDL phenotype.

In this study, we have interrogated the SDL network of *CDC28*. Our data indicate that the underlying cause for the SDL phenotype is likely to be heterogeneous and dependent on the overexpressed gene. For instance, overexpression of a number of genes involved in the organization and orientation of the mitotic spindle revealed that overexpression of *FIN1* significantly altered the assembly of the mitotic spindle in the *cdc28-as1* mutant. Overexpression of several other genes did not result in an obvious defect in *cdc28-as1* mutant cells compared with WT cells. This suggests that the spindle defect in the *cdc28-as1* mutant cells might have been too subtle to be observed by microscopy. Alternatively, Cdc28 activity may be required for cell survival in the presence of mitotic spindle problems, which is supported by our previous studies that showed that loss of mitotic checkpoint activity results in lethality of *cdc28-as1* mutants (Enserink *et al.* 2009; Zimmermann *et al.* 2011).

Another reason for dosage lethality can be the activation of certain cell cycle events in the wrong phase of the cell cycle. For example, we found that overexpression of *CLN3* in 1-NM-PP1-treated *cdc28-as1* mutants, which are arrested in early M phase, resulted in increased transcription of two G1/S-specific cyclin genes, *i.e.*, *CLN2* and *CLB6*, which may further disturb synchrony of the cell cycle. In addition, it is possible that the overexpressed Cln3 titrates away Clb cyclins from the Cdc28 holoenzyme; our genetic data indicate that overexpressed Cln3 is indeed able to activate Cdc28 *in vivo*, as demonstrated by rescue of the temperature-sensitive phenotype of the *cdc28-4* mutant by overexpression of *CLN3* (Figure 4B).

Finally, overexpression of a given Cdc28 target may result in competition with other Cdc28 substrates for phosphorylation by Cdc28, thereby disturbing normal cell cycle progression. This is likely to be particularly toxic in the *cdc28-as1* mutant background, in which Cdc28 activity is limiting. Supporting this hypothesis, we found that overexpression of several known Cdc28 targets in the *cdc28-as1* mutant background resulted in the precocious nuclear entry of Whi5 during M phase; Whi5 is normally localized to the cytoplasm in M phase, because it is phosphorylated by Cdc28, leading to its nuclear export by Msn5 (de Bruin *et al.* 2004; Costanzo *et al.* 2004; Wagner *et al.* 2009; Taberner *et al.* 2009). Nuclear reentry of Whi5 only occurs when it is dephosphorylated (Wagner *et al.* 2009). Thus, the SDL phenotype of at least some genes may result from competition with endogenous Cdc28 substrates, thereby effectively functioning as an inhibitor of Cdc28. In addition to deregulation of cell cycle processes and competition for Cdc28 substrates, it is likely that more explanations exist why certain genes induce dosage lethality in *cdc28-as1* mutants.

We believe that the results from our screen may lead to the identification of additional targets and processes controlled by Cdc28, which is needed to better understand how the cell cycle regulates cell proliferation and homeostasis.

## Supplementary Material

Supplemental material is available online at www.g3journal.org/lookup/suppl/doi:10.1534/g3.117.042317/-/DC1.
