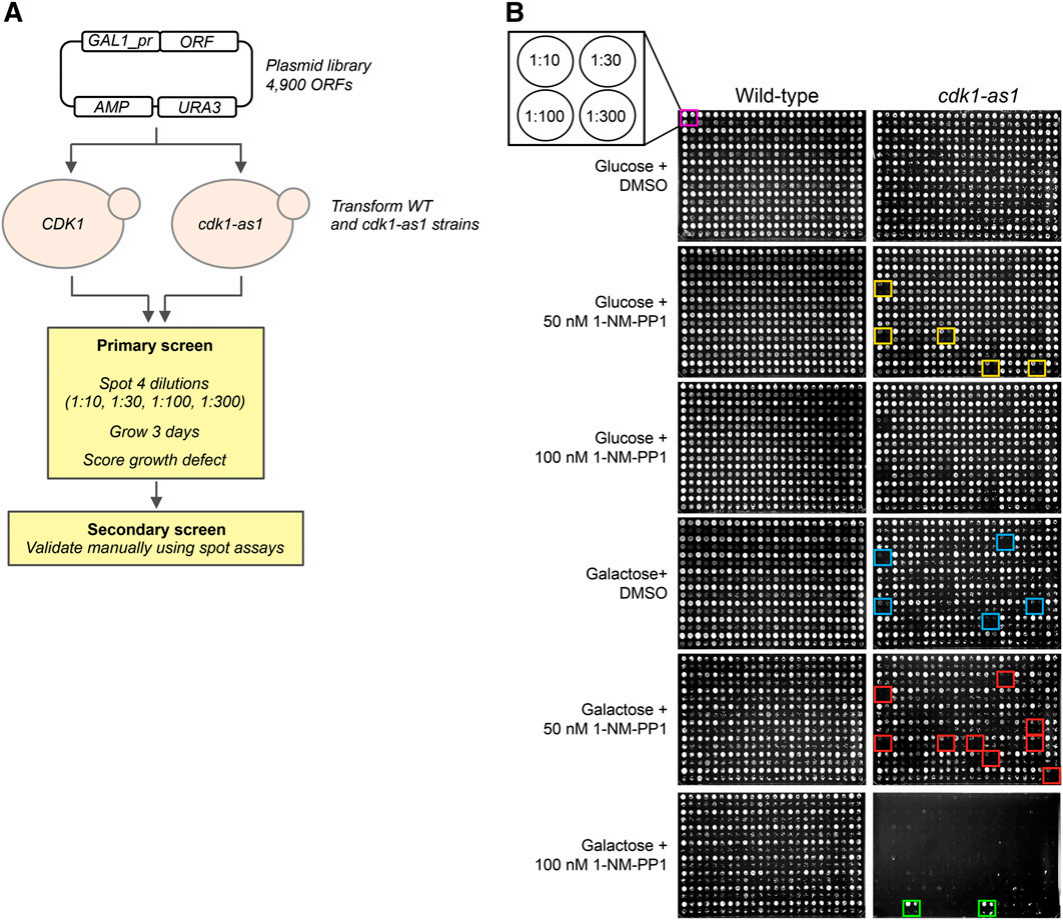# Corrigendum

**DOI:** 10.1534/g3.119.400472

**Published:** 2019-07-31

In the article by C. Zimmermann, I. Garcia, M. Omerzu, P. Chymkowitch, B. Zhang, and J. M. Enserink (*G3: Genes|Genomes|Genetics* 7(6): 1753-1766) entitled “Mapping the Synthetic Dosage Lethality Network of *CDK1/CDC28*,” in Figure 1B on page 1755, a duplicate image was erroneously included for Galactose 50 nM 1-NM-PP1 (Wild-type). Figure 1B has now been corrected.

**Figure 1:**