# Supplementary figures and images for: Mapping the Synthetic Dosage Lethality Network of CDK1/CDC28
Source: G3 (Bethesda). 2017 Apr 18;7(6):1753–66. doi: 10.1534/g3.117.042317 (PMC5473755; doi:10.1534/g3.117.042317)

**A**

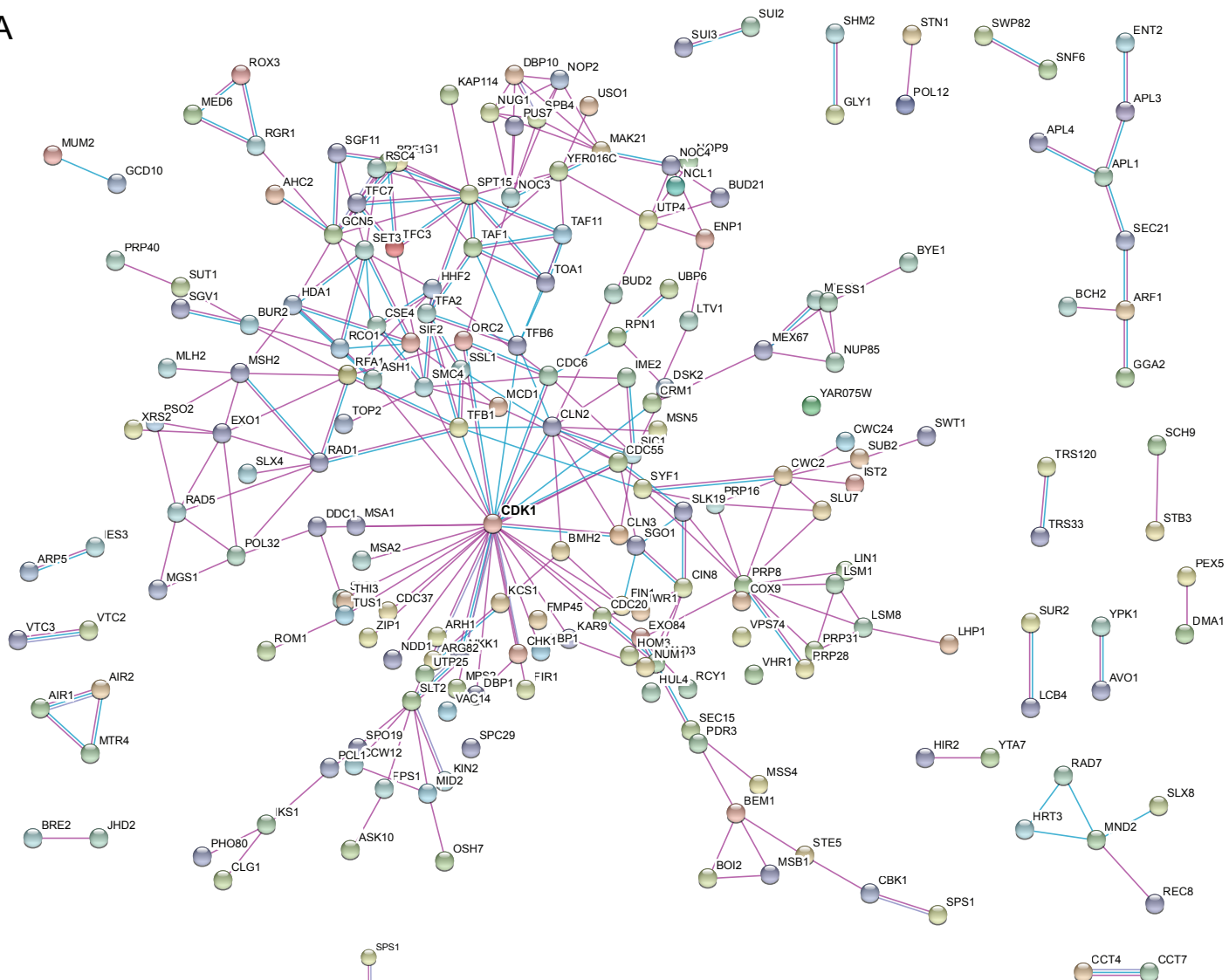

# B

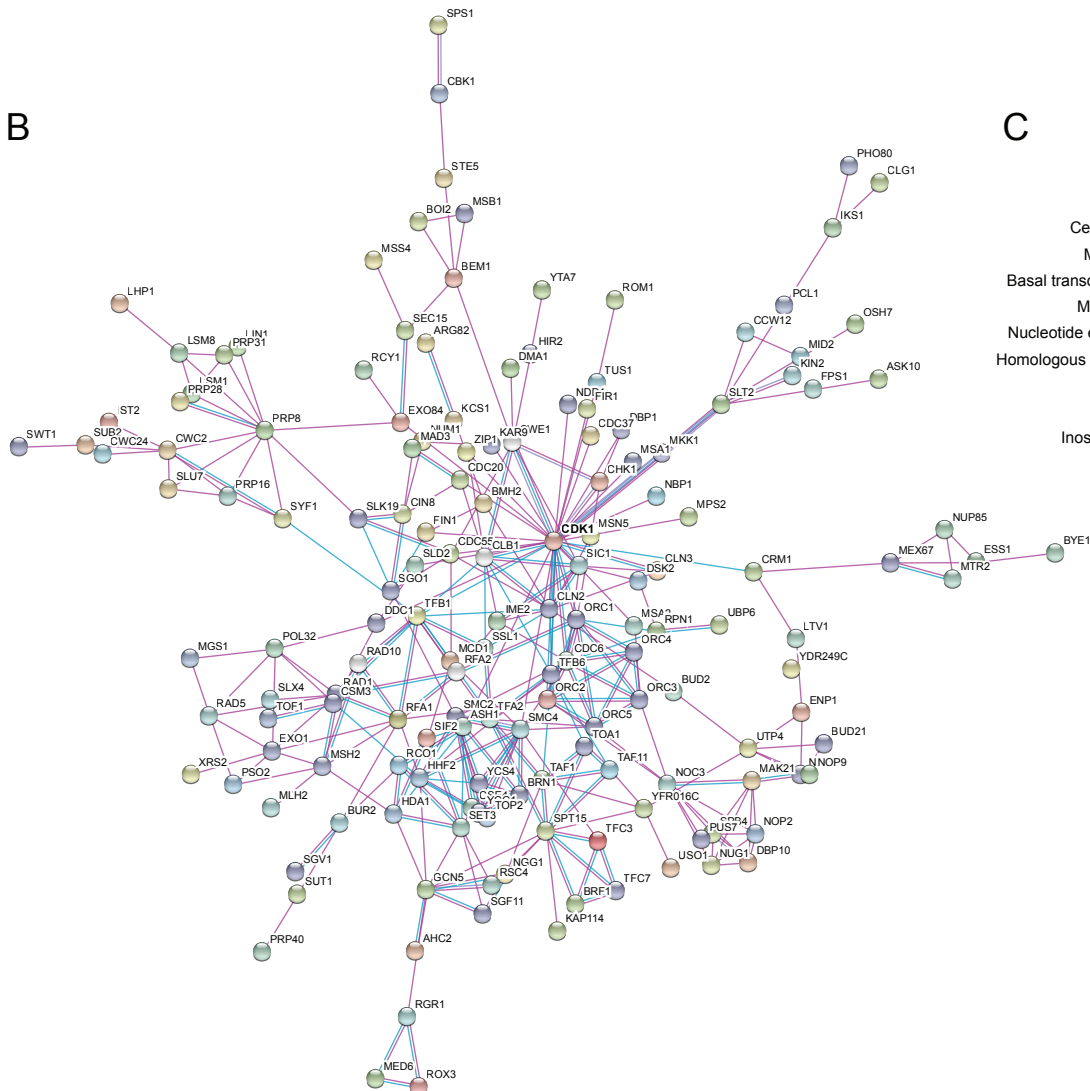

C

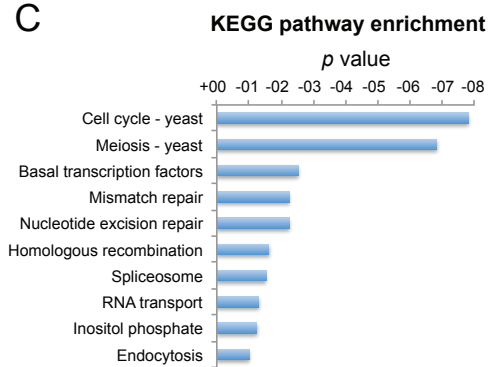

Figure S1. STRING analysis of the SDL network

Supplement: Supplementary file 1 [file 1753FigureS1.pdf]

Supplemental Figure S2

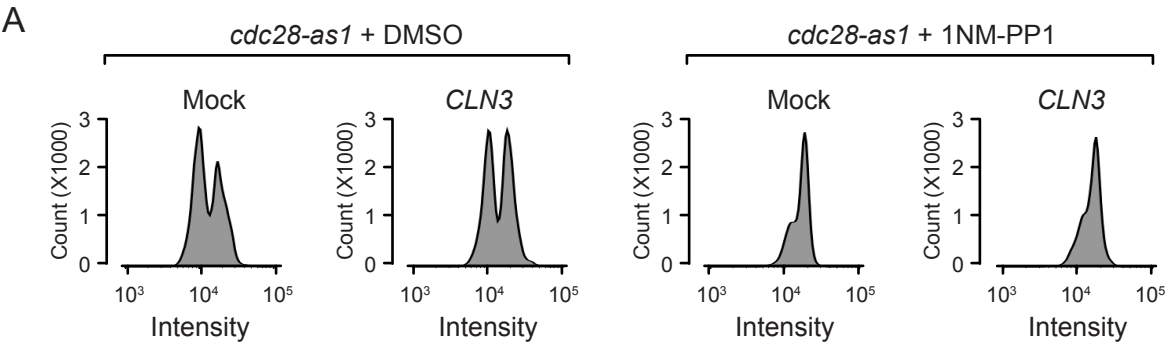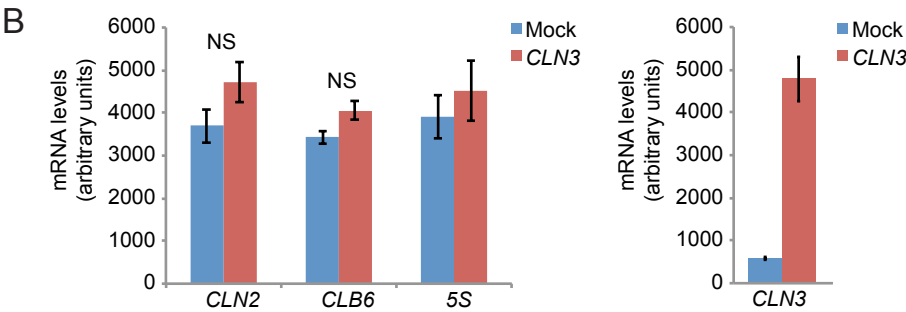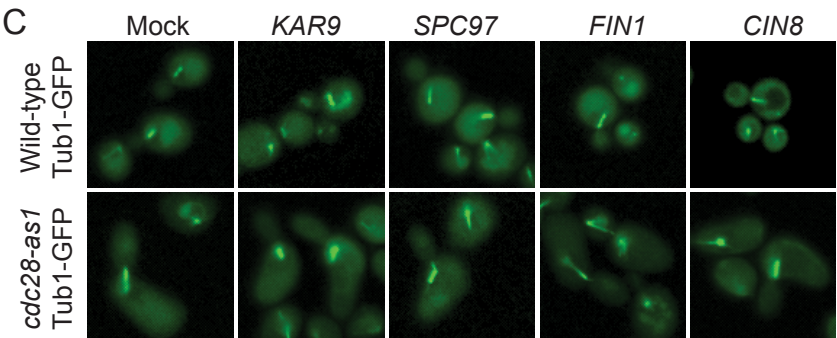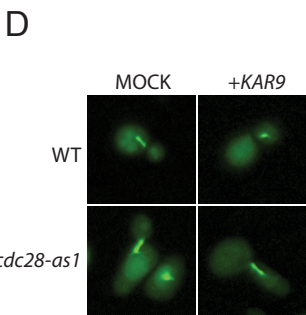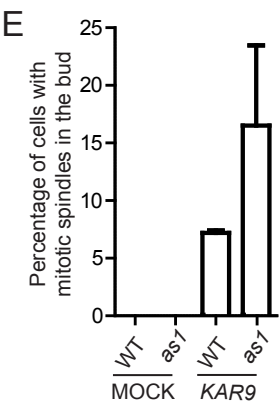

Supplement: Supplementary file 2 [file 1753FigureS2.pdf]

Suppl. Figure S3

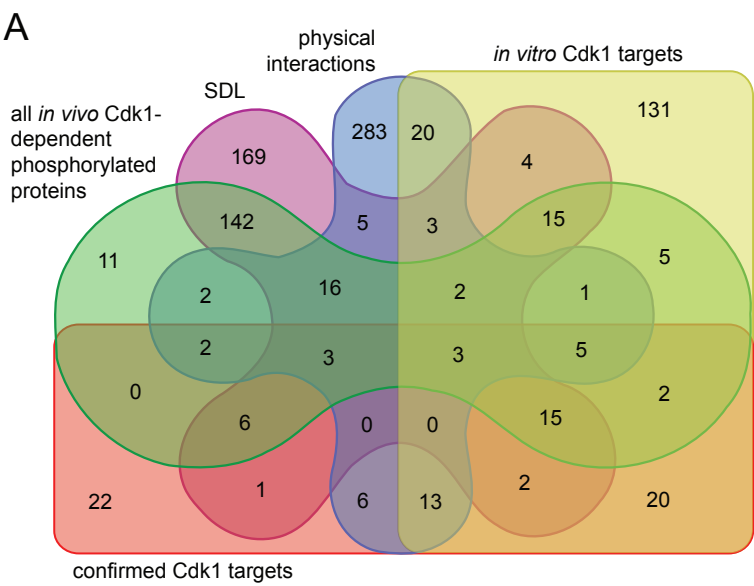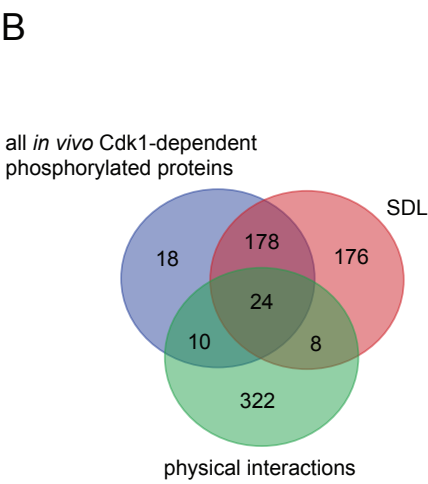

Supplement: Supplementary file 3 [file 1753FigureS3.pdf]

Suppl. Figure S4

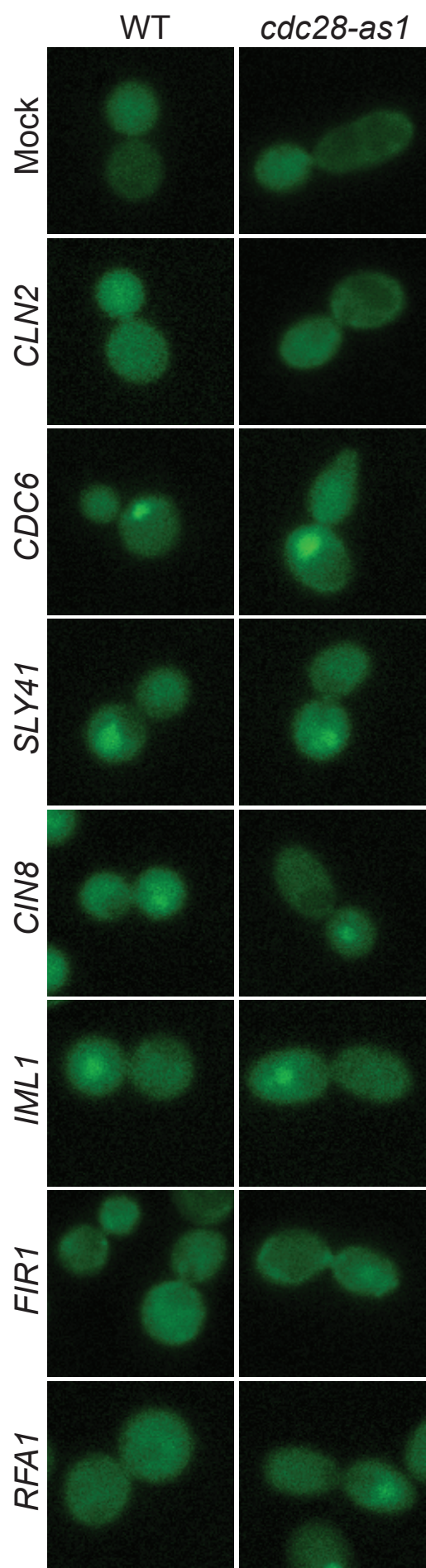

Supplement: Supplementary file 4 [file 1753FigureS4.pdf]
