# Supplementary material for: Mapping the Synthetic Dosage Lethality Network of CDK1/CDC28
Source: G3 (Bethesda). 2017 Apr 18;7(6):1753–66. doi: 10.1534/g3.117.042317 (PMC5473755; doi:10.1534/g3.117.042317)
Supplement: Supplementary file 6 [file 1753TableS2.docx]

**Table S2. Distribution and identity of the ORFs in the Venn diagram shown in Figure 2B.**

| **Class** | **Number of ORFs** | **ORF names** |
| --- | --- | --- |
| - SDL  - negative genetic interactions | 30 | YOL090W YLR386W YJR043C YCR095C YDL003W YNL273W YGR252W YHR082C YMR039C YLR226W YDR285W YGL116W YJL106W YHR030C YNL021W YAR007C YDR326C YNL059C YDR176W YDR168W YGR274C YOR066W YDR017C YDR150W YKR029C YAL040C YOR038C YLR079W YLR032W YDR335W |
| - SDL | 336 | YEL012W YPR161C YML107C YJR022W YPL194W YNL119W YPR072W YOR195W YER114C YDL194W YGR097W YOR162C YOL155C YDR376W YOR262W YFL004W YDL129W YGL233W YEL061C YOR166C YKL049C YLR457C YMR132C YBR030W YBR086C YLR267W YMR195W YLR373C YLR206W YDL113C YKR079C YKL012W YJL051W YDL135C YLR052W YJL111W YNL199C YCL024W YER130C YDR311W YGR266W YPL250C YDR244W YLR135W YHR153C YDL222C YJR052W YIR025W YGL215W YLR372W YDR259C YPL019C YPR169W YNL218W YPL195W YGL241W YGL008C YBR038W YDR006C YIL151C YOL116W YMR212C YMR101C YFL050C YKR010C YLR097C YMR219W YLR005W YIR011C YLR332W YDR416W YLR312C YPL022W YJL050W YER116C YDR243C YER148W YPL047W YOR383C YLR095C YKL186C YOR115C YLR072W YOR071C YER050C YOR307C YPL119C YBL037W YDR504C YGL075C YLL016W YJR092W YOL136C YLR013W YIL056W YML053C YHR058C YMR304W YML015C YOR033C YKR077W YGR077C YKL096W-A YMR075W YDR088C YMR124W YNR063W YKR062W YGR270W YEL046C YKL005C YDR372C YIL079C YKL092C YPL124W YBL024W YGL227W YLR297W YDL169C YLR011W YHR182W YHR185C YER152C YBR068C YPL049C YOR110W YOL001W YGR042W YBL091C YJR119C YER037W YDR249C YJL105W YDL192W YDL025C YBR200W YBR274W YHR138C YER129W YGR091W YMR311C YHL008C YDL080C YDL049C YOR231W YJL107C YOR372C YDL209C YOR284W YCR039C YDR060W YOR188W YDR247W YKR008W YGR211W YLR227C YBR247C YPL130W YHR172W YNL104C YPL237W YBR264C YJL013C YEL043W YLR323C YDL115C YPR144C YDL143W YDR257C YGR070W YOR337W YKR086W YHR187W YBL060W YHL025W YHR205W YNR039C YKL183W YHR075C YIL085C YDR369C YNL289W YLR241W YBL046W YER032W YFL027C YLR429W YOR124C YLR453C YHR001W YJL103C YBL103C YPR143W YKL108W YJL204C YLR002C YMR133W YDR082W YBR199W YPL269W YMR204C YDR407C YER060W YMR137C YCL055W YDR251W YKR027W YNL061W YFL049W YCR005C YJL049W YLR035C YDL175C YKL126W YBL005W YCR082W YDR132C YOR078W YMR302C YLR015W YIL091C YLR086W YGR246C YJR138W YIL157C YOR171C YCR032W YGR218W YJR091C YPR185W YNL314W YDR387C YDR169C YDL031W YMR139W YBR148W YBL033C YDR124W YOR194C YER006W YBR060C YPR021C YER049W YJL124C YKR097W YPR029C YHR165C YNL062C YKL185W YLR096W YOR073W YDR324C YAL001C YOR243C YFL002C YLL043W YKL143W YER156C YDR390C YNL300W YKR041W YJR042W YJR007W YDR085C YKL105C YDR099W YGR146C YNL287W YDR173C YPL169C YDR103W YAR050W YMR276W YJL058C YFR010W YNL161W YNL233W YJL057C YGL250W YCR016W YJL129C YBR102C YML099C YOR352W YHR158C YLR071C YJL031C YLR425W YDL131W YNL088W YDL084W YML082W YCR076C YOR315W YKR096W YFL010C YLR082C YIR023W YHR115C YOR065W YBL035C YEL025C YNL077W YOL028C YPL160W YDR208W YBL093C YGL190C YOR101W YJR017C YHR156C YDR191W YLR110C YHR072W YCL037C YNL103W YOR009W YPR007C YNL030W YJL194W YHR027C YBR057C YDL051W YGR191W YPL256C YDR130C YDR523C YJR005W YOR367W YJL089W YJL010C YOL078W YJL148W YDR297W YML086C YBR103W |
| - negative genetic interactions | 217 | YDL225W YPR046W YDR389W YMR094W YBR245C YDR318W YIL126W YPL015C YDR289C YBR164C YHR178W YMR060C YKL025C YGL058W YHR166C YFR052W YOR027W YCR066W YFL029C YHR119W YPL018W YHR141C YER052C YFR019W YMR055C YKL101W YDR320C YER070W YPL153C YOL012C YNL330C YBR023C YLL002W YPR069C YNL064C YDR260C YMR080C YMR223W YDL017W YIL146C YKL048C YGL003C YMR106C YLR218C YOR026W YDR392W YGR232W YKR054C YDR388W YAL024C YDL108W YHL007C YML094W YER151C YGL094C YLR384C YOR258W YER164W YGL246C YMR153W YKL022C YLR027C YCR084C YDR224C YJL030W YHR200W YPL101W YAR002W YMR001C YOR196C YNL250W YBL008W YKR101W YLR210W YPR085C YDL020C YDL160C YDR174W YCL002C YCR063W YGR054W YGL086W YOR141C YDR113C YML032C YCR009C YBL051C YLR442C YNR051C YGL244W YOL115W YGL240W YGR184C YPR057W YMR300C YPR131C YLR337C YLR418C YGL087C YMR205C YPR089W YER008C YBR126C YOR291W YBL007C YPL017C YDR159W YPR068C YMR224C YPR045C YJL168C YDL047W YIR006C YBR087W YKL213C YKL184W YBR136W YPR120C YGL043W YBR098W YMR186W YDR507C YIL030C YDR145W YDL074C YPR174C YBR036C YGL178W YER095W YOR144C YDR245W YFR012W YNL097C YOL004W YAR015W YHL022C YGL175C YGL057C YAL011W YMR021C YHR059W YOL006C YKL033W YLR176C YDR227W YDL042C YLR329W YDR293C YDR448W YGL216W YKL113C YPR191W YNL189W YDL226C YCL061C YPR141C YER021W YER155C YAR014C YFR027W YBR088C YPL127C YDL002C YCL029C YHR025W YPL008W YGR188C YGL073W YLR357W YJL115W YDL155W YPL055C YML124C YIR021W YMR144W YBL088C YHR014W YGR092W YGL237C YMR078C YPL031C YLR234W YNL107W YDR432W YPR119W YER068W YNL225C YLR055C YAL021C YDR469W YLR089C YGR063C YDL190C YMR190C YKR048C YOL108C YLR360W YOR123C YNL197C YDL056W YGR125W YML103C YPR135W YDL140C YJR075W YPL129W YGL013C YKL041W YBR228W YMR048W YKL054C YPL139C YNR018W YMR167W YKL139W YGR109C YDR440W |
