# Supplementary material for: Mapping the Synthetic Dosage Lethality Network of CDK1/CDC28
Source: G3 (Bethesda). 2017 Apr 18;7(6):1753–66. doi: 10.1534/g3.117.042317 (PMC5473755; doi:10.1534/g3.117.042317)
Supplement: Supplementary file 7 [file 1753TableS3.docx]

**Table S3. Distribution and identity of the ORFs in the Venn diagrams shown in Figure 2C and D.**

| **Class** | **Number of ORFs** | **ORF names** |
| --- | --- | --- |
| - SDL  - SGA | 9 | YNL059C YDR176W YLR386W YHR082C YDR150W YHR030C YKR029C YDR326C YDR335W |
| - SDL | 357 | YLR429W YFL027C YER032W YOR124C YLR453C YEL012W YJL103C YHR001W YPR161C YPR143W YBL103C YML107C YKL108W YJR022W YPL194W YJL204C YNL119W YLR002C YPR072W YMR133W YOR195W YER114C YDR082W YOL090W YDL194W YGR097W YPL269W YBR199W YOR162C YMR204C YER060W YDR407C YOL155C YDR376W YOR262W YFL004W YDL129W YGL233W YEL061C YOR166C YKL049C YMR137C YLR457C YMR132C YCL055W YBR030W YBR086C YDR251W YNL061W YKR027W YFL049W YLR267W YCR005C YMR195W YJL049W YLR035C YLR373C YLR206W YJR043C YDL113C YDL175C YKL126W YKR079C YKL012W YBL005W YJL051W YDL135C YLR052W YDR132C YCR082W YJL111W YNL199C YOR078W YMR302C YLR015W YCL024W YIL091C YER130C YDR311W YLR086W YGR266W YPL250C YGR246C YJR138W YDR244W YDR168W YLR135W YIL157C YOR171C YHR153C YCR032W YDL222C YGR218W YGR274C YJR052W YIR025W YPR185W YJR091C YGL215W YLR372W YDR259C YNL314W YPL019C YDR387C YPR169W YNL218W YDR169C YPL195W YGL241W YMR139W YDL031W YBR148W YBL033C YGL008C YBR038W YDR006C YDL003W YIL151C YDR124W YOR194C YOL116W YMR212C YMR101C YFL050C YKR010C YER006W YLR097C YMR219W YBR060C YPR021C YLR005W YKR097W YJL124C YER049W YIR011C YLR332W YPR029C YDR416W YHR165C YNL062C YLR312C YKL185W YLR096W YPL022W YOR073W YJL050W YDR324C YOR066W YER116C YDR243C YAL001C YOR243C YNL273W YER148W YPL047W YOR383C YLR095C YFL002C YLL043W YKL186C YOR115C YKL143W YLR072W YOR071C YER156C YGR252W YER050C YOR307C YDR390C YPL119C YBL037W YDR504C YNL300W YGL075C YKR041W YLL016W YJR092W YJR042W YOL136C YJR007W YLR013W YDR085C YDR017C YKL105C YIL056W YML053C YDR099W YHR058C YNL287W YGR146C YPL169C YDR173C YMR039C YMR304W YML015C YDR103W YLR226W YAR050W YOR033C YKR077W YMR276W YGR077C YDR285W YJL058C YKL096W-A YFR010W YMR075W YDR088C YNL161W YMR124W YNR063W YKR062W YNL233W YGR270W YEL046C YGL250W YJL057C YCR016W YKL005C YDR372C YIL079C YJL129C YGL116W YKL092C YPL124W YBL024W YGL227W YBR102C YLR297W YDL169C YML099C YOR352W YHR158C YLR011W YLR071C YHR182W YJL106W YHR185C YER152C YJL031C YBR068C YLR425W YPL049C YOR110W YOL001W YDL131W YGR042W YNL088W YBL091C YDL084W YML082W YNL021W YCR076C YJR119C YER037W YOR315W YDR249C YJL105W YKR096W YDL192W YDL025C YFL010C YBR200W YLR082C YBR274W YHR138C YIR023W YOR065W YHR115C YER129W YBL035C YAL040C YGR091W YEL025C YNL077W YMR311C YHL008C YOL028C YDL080C YDL049C YPL160W YOR231W YDR208W YBL093C YOR038C YJL107C YGL190C YOR101W YAR007C YHR156C YJR017C YOR372C YDR191W YDL209C YLR110C YNL103W YCL037C YHR072W YOR284W YOR009W YPR007C YCR039C YNL030W YDR060W YOR188W YHR027C YJL194W YDR247W YKR008W YBR057C YGR211W YDL051W YLR227C YBR247C YGR191W YPL130W YPL256C YDR130C YDR523C YHR172W YLR079W YNL104C YPL237W YBR264C YJL013C YJR005W YEL043W YOR367W YLR323C YDL115C YPR144C YDL143W YDR257C YJL089W YJL010C YGR070W YOL078W YOR337W YKR086W YHR187W YBL060W YHL025W YJL148W YDR297W YLR032W YML086C YHR205W YNR039C YKL183W YBR103W YHR075C YIL085C YDR369C YNL289W YLR241W YBL046W YCR095C |
| - SGA | 97 | YDL225W YPR046W YDR389W YPR120C YGL043W YBR098W YDR318W YDR289C YHR178W YMR060C YKL025C YLR322W YPR174C YBR036C YGL178W YPR044C YDR149C YFR012W YPL018W YHR141C YOL004W YAR015W YER052C YFR019W YAL011W YDR320C YMR021C YER070W YHR059W YKL096C-B YBR023C YDR260C YDR293C YDR448W YGL216W YDR392W YKR054C YDR388W YLR338W YBL062W YCL061C YML094W YER151C YGL094C YOR258W YER155C YAR014C YOL050C YGL246C YMR153W YLR027C YHR025W YDR290W YPL008W YCR084C YGR188C YDL155W YPL055C YML124C YIR021W YMR144W YAR002W YGR092W YGL237C YLR234W YNL107W YDR432W YDL020C YDR174W YCR063W YCL060C YGR054W YGL086W YOR141C YNL225C YLR055C YBL051C YNR051C YGL244W YDL190C YPR057W YMR300C YPR131C YLR337C YBR126C YDL056W YBL007C YPL017C YJR075W YGL013C YPR045C YKL041W YGL217C YBL071W-A YPL139C YKL139W YKL213C |
