# Supplementary material for: Mapping the Synthetic Dosage Lethality Network of CDK1/CDC28
Source: G3 (Bethesda). 2017 Apr 18;7(6):1753–66. doi: 10.1534/g3.117.042317 (PMC5473755; doi:10.1534/g3.117.042317)
Supplement: Supplementary file 9 [file 1753TableS5.docx]

**Table S5. Distribution and identity of the ORFs in the Venn diagram shown in Figure 7A.**

| **Class** | **Number of ORFs** | **ORF names** |
| --- | --- | --- |
| - Confirmed Cdk1 targets  - Physical interactions  - SDL  - In vitro Cdk1 targets  - All in vivo Cdk1-dependent phosphorylated proteins | 3 | YAL040C YLR079W YOR066W |
| - All in vivo Cdk1-dependent phosphorylated proteins  - Physical interactions  - SDL  - In vitro Cdk1 targets | 2 | YLR457C YLR096W |
| - All in vivo Cdk1-dependent phosphorylated proteins  - Physical interactions  - Confirmed Cdk1 targets  - SDL | 3 | YPL269W YJL194W YPL256C |
| - All in vivo Cdk1-dependent phosphorylated proteins  - Confirmed Cdk1 targets  - SDL  - In vitro Cdk1 targets | 15 | YPL194W YOR195W YBR038W YPR160W YBR200W YOR372C YER032W YLR086W YBR060C YKL185W YBR102C YLR425W YDR130C YDR285W YGR270W |
| - All in vivo Cdk1-dependent phosphorylated proteins  - Physical interactions  - Confirmed Cdk1 targets  - In vitro Cdk1 targets | 5 | YJL076W YPL115C YDR146C YNL309W YLR131C |
| - Physical interactions  - Confirmed Cdk1 targets  - SDL  - In vitro Cdk1 targets | 0 |  |
| - All in vivo Cdk1-dependent phosphorylated proteins  - Physical interactions  - SDL | 16 | YDL129W YBR086C YGL008C YBR160W YHR082C YKR077W YPL049C YOR110W YJL013C YDR168W YOR171C YLL043W YNL161W YGL190C YHR027C YJL148W |
| - All in vivo Cdk1-dependent phosphorylated proteins  - SDL  - In vitro Cdk1 targets | 15 | YLR386W YDL113C YJL051W YPL250C YKR010C YJR092W YPL124W YBL024W YDL025C YER129W YOR188W YOR337W YDR176W YJR091C YHR158C |
| - All in vivo Cdk1-dependent phosphorylated proteins  - Confirmed Cdk1 targets  - SDL | 6 | YER114C YEL061C YDR369C YBL046W YDR103W YBL035C |
| - All in vivo Cdk1-dependent phosphorylated proteins  - Physical interactions  - In vitro Cdk1 targets | 1 | YLR319C |
| - All in vivo Cdk1-dependent phosphorylated proteins  - Physical interactions  - Confirmed Cdk1 targets | 2 | YHL007C YKL042W |
| - All in vivo Cdk1-dependent phosphorylated proteins  - Confirmed Cdk1 targets  - In vitro Cdk1 targets | 2 | YOR083W YIL101C |
| - Physical interactions  - SDL  - In vitro Cdk1 targets | 3 | YJL111W YGL116W YOR315W |
| - Confirmed Cdk1 targets  - SDL  - In vitro Cdk1 targets | 2 | YGL075C YKL108W |
| - Physical interactions  - Confirmed Cdk1 targets  - In vitro Cdk1 targets | 13 | YLR045C YGL003C YMR165C YEL032W YAL024C YDR217C YMR001C YDR113C YHR118C YNL068C YJL157C YLR182W YJL187C |
| - All in vivo Cdk1-dependent phosphorylated proteins  - SDL | 142 | YPR161C YNL119W YPR072W YDL194W YGR097W YFL004W YGL233W YLR267W YLR373C YLR206W YJR043C YKR079C YDL135C YLR052W YER052C YNL199C YCL024W YCR095C YDL222C YJR052W YPL019C YPL195W YDR006C YDL003W YIL151C YMR212C YFL050C YMR219W YLR332W YPL022W YJL050W YER116C YDR243C YNL273W YLR095C YKL186C YLR072W YOR071C YBL037W YLR013W YIL056W YGL162W YHR058C YMR039C YML015C YDR088C YMR124W YKR062W YEL046C YKL005C YDR372C YKL092C YNL095C YGL227W YDL169C YHR182W YBR068C YDR003W YOL001W YBL091C YLR058C YHL008C YMR311C YOR231W YAR007C YDL209C YDR326C YDR060W YKR008W YGR211W YBR247C YLR237W YPL237W YEL043W YLR323C YDL058W YGR070W YBL060W YHR205W YNR039C YFR016C YLR429W YOR124C YBL103C YPR143W YNL059C YJL204C YLR002C YMR204C YDR407C YER060W YMR137C YDR251W YNL061W YDL175C YKL126W YOR078W YIL091C YGR246C YJR138W YGR218W YPR185W YDR229W YDR169C YDL031W YMR139W YER006W YPR021C YER049W YKL143W YDR390C YJR007W YDR017C YKL105C YNL287W YDR173C YJL058C YFR010W YNL233W YJL057C YJL129C YOR352W YLR071C YDL131W YDR150W YNL088W YDL084W YFL010C YLR082C YKR029C YPL160W YDR208W YOR101W YNL103W YCL037C YDL051W YGR191W YJR005W YOR367W YOL078W YLR032W YBR103W |
| - All in vivo Cdk1-dependent phosphorylated proteins  - Physical interactions | 2 | YCR088W YER111C |
| - All in vivo Cdk1-dependent phosphorylated proteins  - In vitro Cdk1 targets | 5 | YOL070C YPR174C YDR293C YLL021W YKR095W |
| - Physical interactions  - SDL | 5 | YHR030C YCR016W YOR038C YNL030W YDR335W |
| - SDL  - In vitro Cdk1 targets | 4 | YCL055W YLR035C YKR097W YIR023W |
| - Confirmed Cdk1 targets  - SDL | 1 | YDR082W |
| - Physical interactions  - In vitro Cdk1 targets | 20 | YGR035C YKL048C YPL155C YOR127W YDL153C YDR439W YCL051W YOR075W YBR135W YDR507C YLR187W YKR091W YIL050W YPR141C YJL115W YGR092W YML065W YDR093W YCL014W YJR059W |
| - Physical interactions  - Confirmed Cdk1 targets | 6 | YHR166C YMR199W YDL106C YPL127C YNL225C YGR109C |
| - Confirmed Cdk1 targets  - In vitro Cdk1 targets | 20 | YLR183C YKR089C YHR164C YNL042W YDL220C YPR175W YIL106W YCR065W YFR046C YOR058C YJL092W YDR356W YPL267W YFR027W YML027W YDR501W YDR001C YDR379W YMR036C YER041W |
| - All in vivo Cdk1-dependent phosphorylated proteins | 11 | YBR130C YMR086W YFL014W YLR257W YHR132W-A YGR008C YOL145C YNL106C YKR084C YBR059C YOR042W |
| - SDL | 169 | YEL012W YML107C YJR022W YOR232W YOL090W YOR162C YOL155C YDR376W YOR262W YOR166C YKL049C YMR132C YBR030W YJR036C YMR195W YKL012W YER130C YDR311W YGR266W YDR244W YLR135W YHR153C YIR025W YGL215W YLR372W YDR259C YPR169W YNL218W YGL241W YOL116W YMR101C YLR097C YLR005W YIR011C YBR255C-A YDR416W YLR312C YER148W YPL047W YOR383C YOR115C YGR252W YER050C YOR307C YPL119C YDR504C YLL016W YHR108W YOL136C YML053C YMR304W YLR226W YOR033C YGR077C YKL096W-A YMR075W YNR063W YIL079C YLR297W YLR011W YJL106W YHR185C YER152C YGR042W YNL021W YJR119C YER037W YDR249C YJL105W YDL192W YBR274W YHR138C YGR091W YDL049C YDL080C YJL107C YOR284W YCR039C YDR247W YLR227C YPL130W YHR172W YNL104C YBR264C YDL115C YPR144C YDL143W YDR257C YKR086W YHR187W YHL025W YKL183W YIL085C YHR075C YNL289W YLR241W YFL027C YLR453C YHR001W YJL103C YMR133W YBR199W YKR027W YFL049W YCR005C YJL049W YBL005W YCR082W YDR132C YMR302C YLR015W YIL157C YCR032W YGR274C YNL314W YDR387C YBR148W YBL033C YDR124W YOR194C YJL124C YPR029C YHR165C YNL062C YOR073W YDR324C YAL001C YOR243C YFL002C YER156C YNL300W YDL151C YKR041W YJR042W YDR085C YDR099W YGR146C YPL169C YAR050W YMR276W YGL250W YML099C YJL031C YDL067C YML082W YCR076C YKR096W YHR115C YOR065W YEL025C YNL077W YOL028C YBL093C YHR156C YJR017C YDR191W YPR113W YLR110C YHR072W YOR009W YPR007C YBR057C YPL103C YJR102C YDR523C YJL089W YJL010C YDR297W YML086C |
| - Physical interactions | 283 | YBR118W YDR471W YLR025W YFR034C YLR299W YHR090C YDR418W YJL191W YBL003C YKR080W YGR085C YLR449W YEL009C YDL007W YJR078W YBR010W YDR155C YFL029C YDL055C YPR080W YGR240C YHR141C YNL152W YPR187W YPR086W YHL015W YFR019W YBR072W YBR279W YGR214W YOL039W YNL209W YDR037W YER059W YNL178W YML074C YNL194C YNL064C YLL013C YMR080C YOR198C YML055W YIL131C YLR340W YJL130C YLR212C YLR127C YLR448W YDL082W YNL301C YDL108W YBR189W YLR258W YOR136W YCR012W YNL096C YLR075W YDR385W YDR012W YJL095W YDR381W YJR090C YPL266W YOR275C YGL245W YOL072W YIL022W YER115C YFL009W YLR263W YGL147C YLR141W YGR031W YGR159C YDL185W YMR012W YGL122C YEL013W YJL177W YPL048W YOR259C YLR227W-B YML063W YGL030W YJR076C YGR217W YLR406C YMR319C YML062C YBR029C YPL079W YDL136W YAL003W YBL008W YFR028C YOR043W YBR046C YLR210W YKL023W YOR117W YDR064W YBR111W-A YOR092W YFR004W YGR034W YLR321C YIL018W YGL048C YGL166W YIR031C YML026C YGL123W YGL244W YPR163C YHL001W YOR312C YPR132W YLR153C YPL198W YNL311C YCR087C-A YLR029C YIL107C YLR418C YMR205C YHR032W YHR120W YLL024C YKL060C YNL188W YDR328C YPL240C YKR056W YOR368W YHR021C YLL045C YAL038W YGR155W YDL132W YIL069C YOR354C YOR182C YOL127W YKL211C YJR127C YJR045C YPR120C YBR009C YGL135W YLL039C YBR048W YGR209C YER012W YJR009C YGR148C YLR085C YGL009C YDR222W YJL094C YEL026W YOR133W YGL178W YLR249W YAL058W YKL152C YDR212W YKR025W YBR031W YOR251C YLR410W YOR032C YDR447C YKR059W YGL154C YOR063W YOR062C YML059C YOR369C YAL011W YLR043C YNL304W YBR025C YFR032C-A YMR181C YJR123W YOR234C YJR094W-A YKL172W YLR185W YPL184C YNL189W YJR041C YNL298W YAL035W YGL066W YOL040C YDL083C YDR394W YER125W YCL061C YIL133C YER021W YDR382W YPL249C YAL005C YPL131W YLR259C YNL069C YDR322C-A YDR500C YKL161C YER056C-A YKL145W YFR030W YDL126C YLR061W YOR362C YBL039C YBR079C YLR044C YDL155W YFR015C YDR334W YKL035W YGR027C YBR181C YJL034W YDR301W YLR325C YGR250C YMR207C YIL094C YBR044C YPL031C YGL103W YJL098W YNL107W YGR108W YKL081W YPR119W YKR092C YMR309C YBR127C YDL208W YLR310C YPL221W YOR008C YLR106C YDL014W YPL093W YMR277W YML109W YOR326W YLR180W YOL139C YKR048C YHR099W YFR050C YNL302C YPL020C YNL113W YOR123C YCR028C-A YDL229W YNL031C YNL197C YBL027W YDL056W YJL165C YLR167W YBL092W YER117W YDL140C YOR204W YPR107C YNL016W YER165W YGL016W YHR203C YPR159W YJR082C YHR089C YLR150W |
| - In vitro Cdk1 targets | 131 | YDR123C YPL150W YPL255W YDR389W YKR090W YOR178C YDR348C YIL135C YPR018W YNL058C YKL168C YIL122W YHR098C YDR134C YJL084C YLR455W YHR216W YKL223W YEL065W YNL339C YOR037W YPR111W YJR141W YLR303W YER098W YNL186W YGL097W YLR307W YML119W YOL058W YBL014C YNL257C YKR078W YDL070W YPL209C YDL174C YNL284C-A YOL100W YDR034C-C YOR098C YHR200W YLR006C YBL105C YMR291W YPR171W YGL124C YAR002W YGR014W YHR149C YKL043W YDL089W YDR027C YAL020C YNL271C YOR001W YOR081C YMR005W YDR042C YLR009W YAL019W YOL092W YCL027W YML091C YJL060W YDR223W YMR129W YDL239C YNL272C YMR155W YIL140W YER008C YGR276C YBL007C YDR239C YER167W YDL189W YBR098W YJR033C YBR138C YOR249C YHL022C YDR052C YLR219W YJR083C YLR238W YIL112W YDR227W YDR097C YGL216W YER158C YMR241W YGR296W YHL050C YML083C YBL013W YLR401C YGR221C YNL102W YNR047W YLR278C YGR186W YLL003W YAL028W YNL278W YGL235W YHL035C YPL073C YOR014W YLR394W YDR330W YAL031C YLL008W YLR223C YAR003W YML034W YOL036W YJL199C YKL129C YMR190C YLR190W YLR430W YJR054W YOL125W YPR030W YGR238C YIL031W YOR104W YNL321W YKL116C YOR177C YHR159W |
| - Confirmed Cdk1 targets | 22 | YDL225W YDL028C YHR152W YPL153C YLR102C YKL052C YJR021C YBL085W YMR153W YKL022C YBR156C YGL175C YGL113W YDR451C YCL063W YER155C YLR314C YDR310C YOR373W YBL084C YAR019C YJR089W |
