# Supplementary material for: Mapping the Synthetic Dosage Lethality Network of CDK1/CDC28
Source: G3 (Bethesda). 2017 Apr 18;7(6):1753–66. doi: 10.1534/g3.117.042317 (PMC5473755; doi:10.1534/g3.117.042317)
Supplement: Supplementary file 10 [file 1753TableS6.docx]

**Table S6. Distribution and identity of the ORFs in the Venn diagram shown in Figure 7B.**

| **Class** | **Number of ORFs** | **ORF names** |
| --- | --- | --- |
| - All in vivo Cdk1-dependent phosphorylated proteins  - Physical interactions  - SDL | 24 | YDL129W YLR457C YBR086C YGL008C YBR160W YHR082C YKR077W YPL049C YOR110W YJL013C YPL269W YDR168W YOR171C YLR096W YLL043W YNL161W YAL040C YGL190C YHR027C YJL194W YPL256C YLR079W YJL148W YOR066W |
| - All in vivo Cdk1-dependent phosphorylated proteins  - SDL | 178 | YPR161C YPL194W YNL119W YPR072W YOR195W YER114C YDL194W YGR097W YFL004W YGL233W YEL061C YLR267W YLR373C YLR206W YLR386W YDL113C YJR043C YKR079C YJL051W YDL135C YLR052W YER052C YNL199C YCL024W YPL250C YCR095C YDL222C YJR052W YPL019C YPL195W YDR006C YBR038W YDL003W YIL151C YMR212C YFL050C YKR010C YMR219W YLR332W YPL022W YJL050W YER116C YDR243C YNL273W YLR095C YKL186C YLR072W YOR071C YBL037W YJR092W YLR013W YIL056W YGL162W YHR058C YMR039C YML015C YDR285W YDR088C YMR124W YKR062W YGR270W YEL046C YKL005C YDR372C YKL092C YNL095C YPL124W YBL024W YGL227W YDL169C YHR182W YBR068C YDR003W YOL001W YPR160W YBL091C YLR058C YDL025C YBR200W YER129W YHL008C YMR311C YOR231W YAR007C YOR372C YDL209C YDR326C YDR060W YOR188W YKR008W YGR211W YBR247C YLR237W YPL237W YEL043W YLR323C YDL058W YGR070W YOR337W YBL060W YHR205W YNR039C YFR016C YDR369C YBL046W YER032W YLR429W YOR124C YBL103C YPR143W YNL059C YJL204C YLR002C YMR204C YDR407C YER060W YDR176W YMR137C YDR251W YNL061W YDL175C YKL126W YOR078W YIL091C YLR086W YGR246C YJR138W YGR218W YJR091C YPR185W YDR229W YDR169C YDL031W YMR139W YER006W YBR060C YPR021C YER049W YKL185W YKL143W YDR390C YJR007W YDR017C YKL105C YNL287W YDR173C YDR103W YJL058C YFR010W YNL233W YJL057C YJL129C YBR102C YOR352W YHR158C YLR071C YLR425W YDL131W YDR150W YNL088W YDL084W YFL010C YLR082C YKR029C YBL035C YPL160W YDR208W YOR101W YNL103W YCL037C YDL051W YGR191W YDR130C YJR005W YOR367W YOL078W YLR032W YBR103W |
| - All in vivo Cdk1-dependent phosphorylated proteins  - Physical interactions | 10 | YCR088W YJL076W YHL007C YPL115C YDR146C YNL309W YKL042W YLR131C YER111C YLR319C |
| - Physical interactions  - SDL | 8 | YJL111W YGL116W YHR030C YCR016W YOR315W YOR038C YNL030W YDR335W |
| - All in vivo Cdk1-dependent phosphorylated proteins | 18 | YBR130C YOR083W YMR086W YFL014W YLR257W YOL070C YHR132W-A YGR008C YIL101C YOL145C YPR174C YNL106C YDR293C YKR084C YBR059C YLL021W YKR095W YOR042W |
| - SDL | 176 | YEL012W YML107C YJR022W YOR232W YOL090W YOR162C YOL155C YDR376W YOR262W YOR166C YKL049C YMR132C YBR030W YJR036C YMR195W YKL012W YER130C YDR311W YGR266W YDR244W YLR135W YHR153C YIR025W YGL215W YLR372W YDR259C YPR169W YNL218W YGL241W YOL116W YMR101C YLR097C YLR005W YIR011C YBR255C-A YDR416W YLR312C YER148W YPL047W YOR383C YOR115C YGR252W YER050C YOR307C YPL119C YDR504C YGL075C YLL016W YHR108W YOL136C YML053C YMR304W YLR226W YOR033C YGR077C YKL096W-A YMR075W YNR063W YIL079C YLR297W YLR011W YJL106W YHR185C YER152C YGR042W YNL021W YJR119C YER037W YDR249C YJL105W YDL192W YBR274W YHR138C YGR091W YDL049C YDL080C YJL107C YOR284W YCR039C YDR247W YLR227C YPL130W YHR172W YNL104C YBR264C YDL115C YPR144C YDL143W YDR257C YKR086W YHR187W YHL025W YKL183W YIL085C YHR075C YNL289W YLR241W YFL027C YLR453C YHR001W YJL103C YKL108W YMR133W YDR082W YBR199W YCL055W YKR027W YFL049W YCR005C YJL049W YLR035C YBL005W YCR082W YDR132C YMR302C YLR015W YIL157C YCR032W YGR274C YNL314W YDR387C YBR148W YBL033C YDR124W YOR194C YJL124C YKR097W YPR029C YHR165C YNL062C YOR073W YDR324C YAL001C YOR243C YFL002C YER156C YNL300W YDL151C YKR041W YJR042W YDR085C YDR099W YGR146C YPL169C YAR050W YMR276W YGL250W YML099C YJL031C YDL067C YML082W YCR076C YKR096W YIR023W YHR115C YOR065W YEL025C YNL077W YOL028C YBL093C YHR156C YJR017C YDR191W YPR113W YLR110C YHR072W YOR009W YPR007C YBR057C YPL103C YJR102C YDR523C YJL089W YJL010C YDR297W YML086C |
| - Physical interactions | 322 | YBR118W YDR471W YLR025W YFR034C YLR299W YHR090C YDR418W YJL191W YBL003C YKR080W YLR045C YGR085C YLR449W YEL009C YHR166C YDL007W YJR078W YBR010W YDR155C YFL029C YDL055C YPR080W YGR240C YHR141C YNL152W YPR187W YPR086W YHL015W YFR019W YBR072W YBR279W YGR214W YOL039W YNL209W YDR037W YGR035C YER059W YNL178W YML074C YNL194C YNL064C YLL013C YMR080C YOR198C YKL048C YGL003C YMR165C YML055W YIL131C YLR340W YJL130C YEL032W YLR212C YLR127C YPL155C YLR448W YDL082W YNL301C YAL024C YDL108W YBR189W YLR258W YOR136W YCR012W YOR127W YNL096C YLR075W YDR385W YDL153C YDR012W YJL095W YDR439W YDR381W YJR090C YPL266W YOR275C YGL245W YOL072W YIL022W YER115C YFL009W YLR263W YGL147C YDR217C YLR141W YGR031W YGR159C YDL185W YMR012W YGL122C YEL013W YJL177W YPL048W YOR259C YLR227W-B YML063W YGL030W YJR076C YMR001C YGR217W YLR406C YMR319C YML062C YBR029C YPL079W YDL136W YAL003W YBL008W YFR028C YOR043W YBR046C YLR210W YKL023W YOR117W YDR064W YBR111W-A YOR092W YFR004W YGR034W YLR321C YIL018W YGL048C YGL166W YIR031C YML026C YDR113C YGL123W YGL244W YPR163C YHL001W YOR312C YPR132W YLR153C YMR199W YPL198W YNL311C YCR087C-A YLR029C YIL107C YCL051W YLR418C YMR205C YHR032W YHR120W YLL024C YHR118C YKL060C YNL188W YDR328C YPL240C YKR056W YOR368W YHR021C YLL045C YAL038W YGR155W YOR075W YDL132W YBR135W YIL069C YOR354C YOR182C YOL127W YKL211C YJR127C YJR045C YPR120C YBR009C YGL135W YLL039C YBR048W YGR209C YDR507C YER012W YJR009C YGR148C YLR085C YGL009C YDR222W YJL094C YEL026W YLR187W YOR133W YNL068C YGL178W YLR249W YAL058W YKL152C YDR212W YKR025W YBR031W YOR251C YLR410W YOR032C YDR447C YKR059W YGL154C YOR063W YKR091W YOR062C YML059C YOR369C YAL011W YIL050W YLR043C YNL304W YBR025C YFR032C-A YMR181C YJR123W YOR234C YJR094W-A YKL172W YJL157C YLR185W YPL184C YNL189W YJR041C YNL298W YAL035W YGL066W YOL040C YDL083C YDR394W YER125W YCL061C YPR141C YIL133C YER021W YDR382W YDL106C YPL249C YAL005C YPL131W YLR259C YPL127C YNL069C YDR322C-A YDR500C YKL161C YER056C-A YKL145W YFR030W YDL126C YLR061W YOR362C YBL039C YBR079C YLR044C YJL115W YDL155W YFR015C YDR334W YKL035W YGR027C YBR181C YJL034W YGR092W YDR301W YLR325C YGR250C YMR207C YIL094C YBR044C YPL031C YGL103W YJL098W YNL107W YGR108W YKL081W YPR119W YLR182W YKR092C YMR309C YBR127C YDL208W YLR310C YNL225C YPL221W YML065W YOR008C YLR106C YDL014W YPL093W YMR277W YML109W YOR326W YLR180W YOL139C YKR048C YHR099W YDR093W YFR050C YNL302C YPL020C YNL113W YOR123C YCL014W YCR028C-A YDL229W YNL031C YNL197C YBL027W YDL056W YJL165C YLR167W YBL092W YER117W YDL140C YOR204W YPR107C YNL016W YER165W YGL016W YHR203C YJL187C YJR059W YPR159W YJR082C YHR089C YGR109C YLR150W |
