# Supplementary material for: Mapping the Synthetic Dosage Lethality Network of CDK1/CDC28
Source: G3 (Bethesda). 2017 Apr 18;7(6):1753–66. doi: 10.1534/g3.117.042317 (PMC5473755; doi:10.1534/g3.117.042317)
Supplement: Supplementary file 12 [file 1753TableS8.docx]

**Table S8. Distribution and identity of the ORFs in the Venn diagram shown in Figure 7D.**

| **Class** | **Number of ORFs** | **ORF names** |
| --- | --- | --- |
| - All in vivo Cdk1-dependent phosphorylated proteins  - SDL  - In vitro Cdk1 targets | 34 | YPL194W YOR195W YLR457C YLR386W YDL113C YJL051W YPL250C YBR038W YKR010C YJR092W YDR285W YGR270W YPL124W YBL024W YPR160W YDL025C YBR200W YER129W YOR372C YOR188W YOR337W YER032W YDR176W YLR086W YJR091C YBR060C YKL185W YLR096W YBR102C YHR158C YLR425W YAL040C YDR130C YLR079W |
| - All in vivo Cdk1-dependent phosphorylated proteins  - SDL | 167 | YPR161C YNL119W YPR072W YER114C YDL194W YGR097W YDL129W YFL004W YGL233W YEL061C YBR086C YLR267W YLR373C YLR206W YJR043C YKR079C YDL135C YLR052W YER052C YNL199C YCL024W YCR095C YDL222C YJR052W YPL019C YPL195W YGL008C YDR006C YDL003W YIL151C YMR212C YFL050C YMR219W YLR332W YBR160W YPL022W YJL050W YER116C YDR243C YNL273W YLR095C YKL186C YLR072W YOR071C YBL037W YHR082C YLR013W YIL056W YGL162W YHR058C YMR039C YML015C YKR077W YDR088C YMR124W YKR062W YEL046C YKL005C YDR372C YKL092C YNL095C YGL227W YDL169C YHR182W YBR068C YPL049C YDR003W YOL001W YOR110W YBL091C YLR058C YHL008C YMR311C YOR231W YAR007C YDL209C YDR326C YDR060W YKR008W YGR211W YBR247C YLR237W YPL237W YJL013C YEL043W YLR323C YDL058W YGR070W YBL060W YHR205W YNR039C YFR016C YDR369C YBL046W YLR429W YOR124C YBL103C YPR143W YNL059C YJL204C YLR002C YPL269W YMR204C YDR407C YER060W YMR137C YDR251W YNL061W YDL175C YKL126W YOR078W YIL091C YGR246C YDR168W YJR138W YOR171C YGR218W YPR185W YDR229W YDR169C YDL031W YMR139W YER006W YPR021C YER049W YLL043W YKL143W YDR390C YJR007W YDR017C YKL105C YNL287W YDR173C YDR103W YJL058C YFR010W YNL161W YNL233W YJL057C YJL129C YOR352W YLR071C YDL131W YDR150W YNL088W YDL084W YFL010C YLR082C YKR029C YBL035C YPL160W YDR208W YGL190C YOR101W YNL103W YCL037C YHR027C YJL194W YDL051W YGR191W YPL256C YJR005W YOR367W YOL078W YJL148W YLR032W YBR103W |
| - All in vivo Cdk1-dependent phosphorylated proteins  - In vitro Cdk1 targets | 13 | YJL076W YOR083W YPL115C YDR146C YOL070C YNL309W YIL101C YPR174C YDR293C YLR131C YLL021W YLR319C YKR095W |
| - SDL  - In vitro Cdk1 targets | 10 | YJL111W YGL075C YGL116W YKL108W YCL055W YLR035C YKR097W YOR066W YOR315W YIR023W |
| - All in vivo Cdk1-dependent phosphorylated proteins | 15 | YCR088W YBR130C YMR086W YFL014W YHL007C YLR257W YHR132W-A YGR008C YOL145C YNL106C YKL042W YER111C YKR084C YBR059C YOR042W |
| - SDL | 175 | YEL012W YML107C YJR022W YOR232W YOL090W YOR162C YOL155C YDR376W YOR262W YOR166C YKL049C YMR132C YBR030W YJR036C YMR195W YKL012W YER130C YDR311W YGR266W YDR244W YLR135W YHR153C YIR025W YGL215W YLR372W YDR259C YPR169W YNL218W YGL241W YOL116W YMR101C YLR097C YLR005W YIR011C YBR255C-A YDR416W YLR312C YER148W YPL047W YOR383C YOR115C YGR252W YER050C YOR307C YPL119C YDR504C YLL016W YHR108W YOL136C YML053C YMR304W YLR226W YOR033C YGR077C YKL096W-A YMR075W YNR063W YIL079C YLR297W YLR011W YJL106W YHR185C YER152C YGR042W YHR030C YNL021W YJR119C YER037W YDR249C YJL105W YDL192W YBR274W YHR138C YGR091W YDL049C YDL080C YJL107C YOR284W YCR039C YDR247W YLR227C YPL130W YHR172W YNL104C YBR264C YDL115C YPR144C YDL143W YDR257C YKR086W YHR187W YHL025W YKL183W YIL085C YHR075C YNL289W YLR241W YFL027C YLR453C YHR001W YJL103C YMR133W YDR082W YBR199W YKR027W YFL049W YCR005C YJL049W YBL005W YCR082W YDR132C YMR302C YLR015W YIL157C YCR032W YGR274C YNL314W YDR387C YBR148W YBL033C YDR124W YOR194C YJL124C YPR029C YHR165C YNL062C YOR073W YDR324C YAL001C YOR243C YFL002C YER156C YNL300W YDL151C YKR041W YJR042W YDR085C YDR099W YGR146C YPL169C YAR050W YMR276W YGL250W YCR016W YML099C YJL031C YDL067C YML082W YCR076C YKR096W YHR115C YOR065W YEL025C YNL077W YOL028C YBL093C YOR038C YHR156C YJR017C YDR191W YPR113W YLR110C YHR072W YOR009W YPR007C YNL030W YBR057C YPL103C YJR102C YDR523C YJL089W YJL010C YDR297W YDR335W YML086C |
| - In vitro Cdk1 targets | 184 | YDR123C YPL150W YPL255W YDR389W YKR090W YLR183C YOR178C YLR045C YDR348C YIL135C YPR018W YNL058C YKL168C YIL122W YKR089C YHR098C YHR164C YDR134C YJL084C YLR455W YHR216W YKL223W YGR035C YEL065W YNL339C YOR037W YKL048C YGL003C YMR165C YPR111W YEL032W YJR141W YLR303W YER098W YPL155C YNL186W YGL097W YAL024C YLR307W YML119W YOL058W YNL042W YOR127W YBL014C YDL220C YNL257C YDL153C YDR439W YKR078W YDL070W YPL209C YDL174C YDR217C YNL284C-A YOL100W YDR034C-C YOR098C YHR200W YLR006C YBL105C YMR291W YPR171W YGL124C YAR002W YMR001C YGR014W YHR149C YKL043W YDL089W YDR027C YAL020C YNL271C YOR001W YDR113C YOR081C YMR005W YDR042C YLR009W YAL019W YOL092W YCL027W YML091C YJL060W YDR223W YMR129W YDL239C YPR175W YNL272C YMR155W YCL051W YIL106W YIL140W YER008C YGR276C YBL007C YHR118C YDR239C YER167W YDL189W YOR075W YBR135W YBR098W YJR033C YCR065W YDR507C YLR187W YNL068C YBR138C YFR046C YOR249C YHL022C YKR091W YOR058C YJL092W YDR052C YLR219W YDR356W YJR083C YLR238W YIL112W YIL050W YDR227W YJL157C YDR097C YGL216W YER158C YMR241W YGR296W YHL050C YPL267W YML083C YBL013W YLR401C YGR221C YPR141C YNL102W YNR047W YFR027W YLR278C YGR186W YAL028W YLL003W YNL278W YML027W YGL235W YHL035C YJL115W YPL073C YGR092W YOR014W YLR394W YDR330W YDR501W YDR001C YDR379W YLR182W YAL031C YLL008W YML065W YLR223C YAR003W YML034W YOL036W YJL199C YKL129C YMR190C YLR190W YDR093W YMR036C YLR430W YCL014W YJR054W YOL125W YPR030W YGR238C YIL031W YOR104W YNL321W YKL116C YOR177C YJL187C YJR059W YHR159W YER041W |
