# Supplementary material for: Mapping the Synthetic Dosage Lethality Network of CDK1/CDC28
Source: G3 (Bethesda). 2017 Apr 18;7(6):1753–66. doi: 10.1534/g3.117.042317 (PMC5473755; doi:10.1534/g3.117.042317)
Supplement: Supplementary file 13 [file 1753TableS9.docx]

**Table S9. Distribution and identity of the ORFs in the Venn diagram shown in Figure 7E.**

| **Class** | **Number of ORFs** | **ORF names** |
| --- | --- | --- |
| - All in vivo Cdk1-dependent phosphorylated proteins  - Confirmed Cdk1 targets  - SDL | 26 | YER032W YPL194W YOR195W YER114C YPL269W YEL061C YLR086W YBR038W YBR060C YKL185W YDR103W YBR102C YLR425W YPR160W YBR200W YBL035C YAL040C YOR372C YJL194W YPL256C YDR130C YLR079W YDR369C YBL046W YDR285W YGR270W |
| - All in vivo Cdk1-dependent phosphorylated proteins  - SDL | 175 | YLR429W YOR124C YPR161C YPR143W YBL103C YNL059C YJL204C YNL119W YLR002C YPR072W YDL194W YGR097W YMR204C YER060W YDR407C YDR176W YDL129W YFL004W YGL233W YMR137C YLR457C YBR086C YDR251W YNL061W YLR267W YLR373C YLR206W YLR386W YDL175C YDL113C YJR043C YKL126W YKR079C YJL051W YDL135C YLR052W YER052C YNL199C YOR078W YCL024W YIL091C YPL250C YGR246C YCR095C YJR138W YDR168W YOR171C YDL222C YGR218W YJR052W YPR185W YJR091C YDR229W YPL019C YDR169C YPL195W YMR139W YDL031W YGL008C YDR006C YDL003W YIL151C YMR212C YFL050C YKR010C YER006W YMR219W YPR021C YER049W YLR332W YBR160W YLR096W YPL022W YJL050W YER116C YDR243C YNL273W YLR095C YLL043W YKL186C YKL143W YLR072W YOR071C YDR390C YBL037W YJR092W YJR007W YHR082C YLR013W YDR017C YKL105C YIL056W YGL162W YHR058C YNL287W YDR173C YMR039C YML015C YKR077W YJL058C YFR010W YDR088C YNL161W YMR124W YKR062W YNL233W YEL046C YJL057C YKL005C YDR372C YJL129C YKL092C YNL095C YPL124W YBL024W YGL227W YDL169C YOR352W YHR158C YLR071C YHR182W YBR068C YPL049C YDR003W YOL001W YOR110W YDL131W YDR150W YNL088W YBL091C YDL084W YLR058C YDL025C YFL010C YLR082C YKR029C YER129W YHL008C YMR311C YPL160W YOR231W YDR208W YGL190C YOR101W YAR007C YDL209C YDR326C YCL037C YNL103W YDR060W YHR027C YOR188W YKR008W YGR211W YDL051W YBR247C YGR191W YLR237W YPL237W YJL013C YJR005W YEL043W YLR323C YOR367W YDL058W YGR070W YOL078W YOR337W YBL060W YJL148W YLR032W YHR205W YNR039C YFR016C YBR103W |
| - All in vivo Cdk1-dependent phosphorylated proteins  - Confirmed Cdk1 targets | 9 | YIL101C YJL076W YOR083W YKL042W YHL007C YPL115C YLR131C YDR146C YNL309W |
| - Confirmed Cdk1 targets  - SDL | 4 | YKL108W YDR082W YOR066W YGL075C |
| - All in vivo Cdk1-dependent phosphorylated proteins | 19 | YCR088W YBR130C YOL145C YPR174C YMR086W YFL014W YNL106C YDR293C YER111C YLR257W YKR084C YBR059C YOL070C YHR132W-A YLL021W YLR319C YGR008C YKR095W YOR042W |
| - SDL | 181 | YFL027C YLR453C YEL012W YJL103C YHR001W YML107C YJR022W YOR232W YMR133W YOL090W YBR199W YOR162C YOL155C YDR376W YOR262W YOR166C YKL049C YMR132C YCL055W YBR030W YKR027W YFL049W YJR036C YCR005C YMR195W YJL049W YLR035C YKL012W YBL005W YDR132C YCR082W YJL111W YMR302C YLR015W YER130C YDR311W YGR266W YDR244W YLR135W YIL157C YHR153C YCR032W YGR274C YIR025W YGL215W YLR372W YDR259C YNL314W YDR387C YPR169W YNL218W YGL241W YBR148W YBL033C YDR124W YOR194C YOL116W YMR101C YLR097C YLR005W YKR097W YJL124C YIR011C YPR029C YBR255C-A YDR416W YHR165C YNL062C YLR312C YOR073W YDR324C YAL001C YOR243C YER148W YPL047W YOR383C YFL002C YOR115C YER156C YGR252W YER050C YOR307C YPL119C YDR504C YNL300W YDL151C YKR041W YLL016W YHR108W YJR042W YOL136C YDR085C YML053C YDR099W YGR146C YPL169C YMR304W YLR226W YAR050W YOR033C YMR276W YGR077C YKL096W-A YMR075W YNR063W YGL250W YCR016W YIL079C YGL116W YLR297W YML099C YLR011W YJL106W YHR185C YJL031C YER152C YDL067C YGR042W YHR030C YML082W YNL021W YCR076C YJR119C YER037W YOR315W YDR249C YJL105W YKR096W YDL192W YBR274W YHR138C YIR023W YOR065W YHR115C YGR091W YEL025C YNL077W YOL028C YDL049C YDL080C YBL093C YOR038C YJL107C YJR017C YHR156C YPR113W YDR191W YLR110C YHR072W YOR284W YOR009W YPR007C YCR039C YNL030W YDR247W YBR057C YLR227C YPL103C YPL130W YJR102C YHR172W YDR523C YNL104C YBR264C YDL115C YPR144C YDL143W YDR257C YJL089W YJL010C YKR086W YHR187W YHL025W YDR335W YDR297W YML086C YKL183W YIL085C YHR075C YNL289W YLR241W |
| - Confirmed Cdk1 targets | 61 | YDL225W YCR065W YDL028C YLR183C YHR152W YLR045C YHR166C YNL068C YFR046C YKR089C YHR164C YGL175C YOR058C YJL092W YDR356W YPL153C YLR102C YGL003C YMR165C YGL113W YEL032W YKL052C YJL157C YDR451C YJR021C YCL063W YAL024C YPL267W YBL085W YNL042W YDL106C YER155C YFR027W YDL220C YPL127C YMR153W YKL022C YML027W YLR314C YDR217C YDR310C YOR373W YMR001C YBL084C YDR501W YDR001C YDR379W YLR182W YAR019C YNL225C YDR113C YMR199W YJR089W YPR175W YMR036C YIL106W YHR118C YBR156C YJL187C YGR109C YER041W |
