# Supplementary material for: Mapping the Synthetic Dosage Lethality Network of CDK1/CDC28
Source: G3 (Bethesda). 2017 Apr 18;7(6):1753–66. doi: 10.1534/g3.117.042317 (PMC5473755; doi:10.1534/g3.117.042317)
Supplement: Supplementary file 14 [file 1753TableS10.docx]

**Table S10. Distribution and identity of the ORFs in the Venn diagram shown in Figure 7F.**

| **Class** | **Number of ORFs** | **ORF names** |
| --- | --- | --- |
| - SDL  - Confirmed Cdk1 targets  - In vivo Cdk1-dependent protein phosphorylation on [S/T]-P sites | 14 | YER032W YER114C YPL269W YLR086W YBR038W YBR060C YKL185W YDR103W YBR102C YBL035C YJL194W YDR130C YDR369C YBL046W |
| - SDL  - Confirmed Cdk1 targets | 14 | YKL108W YPL194W YOR195W YDR082W YEL061C YOR066W YGL075C YLR425W YPR160W YBR200W YAL040C YOR372C YPL256C YLR079W |
| - SDL  - In vivo Cdk1-dependent protein phosphorylation on [S/T]-P sites | 30 | YOR124C YPR072W YGR097W YMR137C YLR457C YDR006C YMR219W YLR096W YJR092W YKL105C YKR077W YNL161W YMR124W YKR062W YNL233W YKL005C YKL092C YPL124W YBL024W YGL227W YHR158C YBR068C YNL088W YER129W YPL160W YDR326C YCL037C YJL013C YEL043W YNR039C |
| - Confirmed Cdk1 targets | 70 | YDL225W YIL101C YCR065W YDL028C YLR183C YHR152W YLR045C YJL076W YOR083W YHR166C YNL068C YFR046C YHR164C YKR089C YGL175C YOR058C YJL092W YDR356W YPL153C YLR102C YGL003C YMR165C YGL113W YEL032W YKL052C YJL157C YKL042W YDR451C YJR021C YCL063W YAL024C YPL267W YHL007C YBL085W YPL115C YNL042W YDL106C YER155C YLR131C YFR027W YDL220C YPL127C YMR153W YKL022C YLR314C YML027W YDR217C YDR310C YOR373W YMR001C YBL084C YDR146C YDR501W YDR001C YDR379W YAR019C YLR182W YNL225C YDR113C YMR199W YJR089W YPR175W YMR036C YIL106W YHR118C YBR156C YJL187C YGR109C YNL309W YER041W |
| - SDL | 328 | YLR429W YFL027C YLR453C YEL012W YJL103C YHR001W YPR161C YPR143W YBL103C YML107C YJR022W YNL059C YJL204C YNL119W YLR002C YOR232W YMR133W YOL090W YDL194W YBR199W YOR162C YMR204C YER060W YDR407C YOL155C YDR176W YDR376W YOR262W YFL004W YDL129W YGL233W YOR166C YKL049C YMR132C YCL055W YBR086C YBR030W YDR251W YNL061W YKR027W YFL049W YLR267W YJR036C YCR005C YMR195W YJL049W YLR035C YLR373C YLR206W YLR386W YJR043C YDL175C YDL113C YKL126W YKR079C YKL012W YBL005W YJL051W YDL135C YLR052W YDR132C YCR082W YER052C YJL111W YNL199C YOR078W YMR302C YLR015W YCL024W YIL091C YER130C YDR311W YGR266W YPL250C YGR246C YCR095C YJR138W YDR244W YDR168W YLR135W YIL157C YOR171C YHR153C YDL222C YCR032W YGR218W YGR274C YJR052W YIR025W YPR185W YJR091C YGL215W YLR372W YDR259C YDR229W YNL314W YPL019C YDR387C YPR169W YNL218W YDR169C YPL195W YGL241W YMR139W YDL031W YBR148W YBL033C YGL008C YDL003W YIL151C YDR124W YOR194C YOL116W YMR212C YMR101C YFL050C YKR010C YER006W YLR097C YPR021C YLR005W YKR097W YJL124C YER049W YIR011C YLR332W YBR160W YPR029C YBR255C-A YDR416W YHR165C YNL062C YLR312C YPL022W YOR073W YJL050W YDR324C YER116C YDR243C YAL001C YOR243C YNL273W YER148W YPL047W YOR383C YLR095C YFL002C YLL043W YKL186C YOR115C YKL143W YLR072W YOR071C YER156C YGR252W YER050C YOR307C YDR390C YPL119C YDR504C YBL037W YNL300W YDL151C YKR041W YLL016W YHR108W YJR042W YOL136C YJR007W YHR082C YLR013W YDR085C YDR017C YIL056W YML053C YGL162W YDR099W YHR058C YNL287W YGR146C YPL169C YDR173C YMR039C YMR304W YML015C YLR226W YAR050W YOR033C YMR276W YGR077C YDR285W YJL058C YKL096W-A YFR010W YMR075W YDR088C YNR063W YGR270W YJL057C YEL046C YGL250W YCR016W YJL129C YDR372C YIL079C YGL116W YNL095C YLR297W YDL169C YML099C YOR352W YLR071C YLR011W YJL106W YHR182W YHR185C YJL031C YER152C YPL049C YDR003W YDL067C YOR110W YOL001W YDL131W YGR042W YDR150W YHR030C YBL091C YDL084W YML082W YNL021W YCR076C YJR119C YER037W YOR315W YDR249C YJL105W YKR096W YDL192W YLR058C YDL025C YFL010C YLR082C YBR274W YHR138C YIR023W YKR029C YOR065W YHR115C YGR091W YEL025C YNL077W YMR311C YHL008C YOL028C YDL049C YDL080C YOR231W YDR208W YBL093C YOR038C YJL107C YGL190C YOR101W YAR007C YJR017C YHR156C YPR113W YDR191W YDL209C YLR110C YNL103W YHR072W YOR284W YOR009W YPR007C YCR039C YNL030W YDR060W YOR188W YHR027C YDR247W YKR008W YBR057C YGR211W YDL051W YLR227C YPL103C YLR237W YBR247C YGR191W YPL130W YJR102C YDR523C YHR172W YNL104C YPL237W YBR264C YJR005W YOR367W YLR323C YDL115C YDL058W YPR144C YDL143W YDR257C YJL089W YJL010C YGR070W YOL078W YOR337W YKR086W YHR187W YBL060W YHL025W YJL148W YLR032W YDR297W YDR335W YML086C YHR205W YKL183W YFR016C YBR103W YHR075C YIL085C YNL289W YLR241W |
